# Application of Nanotrap technology for high sensitivity measurement of urinary outer surface protein A carboxyl-terminus domain in early stage Lyme borreliosis

**DOI:** 10.1186/s12967-015-0701-z

**Published:** 2015-11-04

**Authors:** Ruben Magni, Benjamin H. Espina, Ketul Shah, Benjamin Lepene, Christine Mayuga, Temple A. Douglas, Virginia Espina, Sally Rucker, Ross Dunlap, Emanuel F. III Petricoin, Mary Frekko Kilavos, Donald M. Poretz, Gilbert R. Irwin, Samuel M. Shor, Lance A. Liotta, Alessandra Luchini

**Affiliations:** George Mason University, Manassas, VA USA; University of Milan, Milan, Italy; Ceres Nanosciences, Manassas, VA USA; Frekko Primary Care, Gaithersburg, MD USA; Care ID, Annandale, VA USA; Novant Health, Manassas, VA USA; Internal Medicine of Northern Virginia, Reston, VA USA

## Abstract

**Objectives:**

Prompt antibiotic treatment of early stage Lyme borreliosis (LB) prevents progression to severe multisystem disease. There is a clinical need to improve the diagnostic specificity of early stage Lyme assays in the period prior to the mounting of a robust serology response. Using a novel analyte harvesting nanotechnology, Nanotrap particles, we evaluated urinary *Borrelia* Outer surface protein A (OspA) C-terminus peptide in early stage LB before and after treatment, and in patients suspected of late stage disseminated LB.

**Method:**

We employed Nanotrap particles to concentrate urinary OspA and used a highly specific anti-OspA monoclonal antibody (mAb) as a detector of the C-terminus peptides. We mapped the mAb epitope to a narrow specific OspA C-terminal domain OspA236-239 conserved across infectious *Borrelia* species but with no homology to human proteins and no cross-reactivity with relevant viral and non-*Borrelia* bacterial proteins. 268 urine samples from patients being evaluated for all categories of LB were collected in a LB endemic area. The urinary OspA assay, blinded to outcome, utilized Nanotrap particle pre-processing, western blotting to evaluate the OspA molecular size, and OspA peptide competition for confirmation.

**Results:**

OspA test characteristics: sensitivity 1.7 pg/mL (lowest limit of detection), % coefficient of variation (CV) = 8 %, dynamic range 1.7–30 pg/mL. Pre-treatment, 24/24 newly diagnosed patients with an erythema migrans (EM) rash were positive for urinary OspA while false positives for asymptomatic patients were 0/117 (Chi squared p < 10^−6^). For 10 patients who exhibited persistence of the EM rash during the course of antibiotic therapy, 10/10 were positive for urinary OspA. Urinary OspA of 8/8 patients switched from detectable to undetectable following symptom resolution post-treatment. Specificity of the urinary OspA test for the clinical symptoms was 40/40. Specificity of the urinary OspA antigen test for later serology outcome was 87.5 % (21 urinary OspA positive/24 serology positive, Chi squared p = 4.072e^−15^). 41 of 100 patients under surveillance for persistent LB in an endemic area were positive for urinary OspA protein.

**Conclusions:**

OspA urinary shedding was strongly linked to concurrent active symptoms (e.g. EM rash and arthritis), while resolution of these symptoms after therapy correlated with urinary conversion to OspA negative.

**Electronic supplementary material:**

The online version of this article (doi:10.1186/s12967-015-0701-z) contains supplementary material, which is available to authorized users.

## Background

Prompt antibiotic treatment of early stage Lyme borreliosis (LB) can prevent progression of the disease from the localized stage to the early and late disseminated stages [1, 2]. Unfortunately, because the clinical presentation can be so varied, early stage disease can be misdiagnosed for a variety of reasons including failure to develop an erythema migrans (EM) rash [3–6], failure of the patient or clinician to recognize an EM rash, if present [4–7], the non-specific nature of early symptoms (fatigue, fever, headache, muscle and joint pains, swollen lymph nodes), and a negative, or ambiguous serology [8–12]. Moreover, even after a single first course of antibiotic therapy, a small but significant percentage of patients and experimentally infected animals can continue to harbor *Borrelia* [13–15]. Thus there is a clinical need to improve the diagnostic specificity of early stage Lyme assays, particularly in the period prior to the mounting of a robust serologic response [8, 10]. In addition, it would be valuable to know with greater certainty whether a first round of therapy is successful or should be repeated because of *Borrelia* persistence [8, 10, 16, 17]. To address these needs we evaluated urinary *Borrelia* Outer surface protein A (OspA) in early stage LB using an analyte harvesting nanotechnology, Nanotrap particles, to achieve high sensitivity [18, 19], coupled with an anti-OspA monoclonal antibody (mAb) which we show herein to recognize a narrow specific OspA C-terminal region, OspA236-239. OspA26-239 sequence is conserved across infectious *Borrelia* species, but does not have sequence homology with human or non-*Borrelia* relevant pathogens.

We selected OspA for urinary monitoring of early stage LB for several reasons including its central role in the early stage of pathogenesis [20], the known shedding of *Borrelia* antigen in the urine of animals infected with *Borrelia burgdorferi* (Bb) [21], and the OspA sequence conservation across *Borrelia* species [22, 23]. OspA is a 273 amino acid protein that folds in an elongated conformation spanning 80 Å from end to end. OspA binds to the surface of the spirochete at the N-terminus via a lipid anchor. The structure consists of 21 consecutive antiparallel β-strands followed by a short α-helix in the C-terminus and can be divided into two discrete domains: a sandwich domain at the N-terminus and a barrel domain at the C-terminus [23]. The OspA barrel domain at the C-terminus is highly conserved across *Borrelia* pathogenic species and plays an important role in Bb induced immune tolerance, induction of the inflammatory response through TLR2 [14, 24], and host immunologic recognition [20]. In this study we focused on the shedding of urinary OspA peptides that contain the critical C-terminus domain. Despite the strong rationale for evaluating *Borrelia* antigens in body fluids for diagnostic purposes in patients with LB, including cerebrospinal fluid and urine [25, 26], the validity of previous immunoassays for *Borrelia* proteins has remained controversial [27], due to questions of specificity and sensitivity. Previous immunoassays for *Borrelia* antigens have employed polyclonal antibodies raised against *Borrelia* culture lysates. These assays may have had limited sensitivity, and may not have been directed at a single specific conserved epitope of OspA lacking sequence homology with human proteins or non-*Borrelia* spirochetes. Attempts to culture *Borrelia* from infected individuals, or to measure *Borrelia* nucleic acid in blood by PCR [28] have indicated that the titer of *Borrelia* spirochetes is very low. In addition, it has been documented that *Borrelia* can be sequestered and persist in joint tissues [17]. Consequently the concentration of OspA antigen in urine is expected to be very low, well below one nanogram per mL. Thus, even though there has been a historical recognition of the value of measuring OspA antigens in body fluids, the immune-epitope specificity and sensitivity of past methods has not been sufficient to address this important question.

**Fig. 1:**
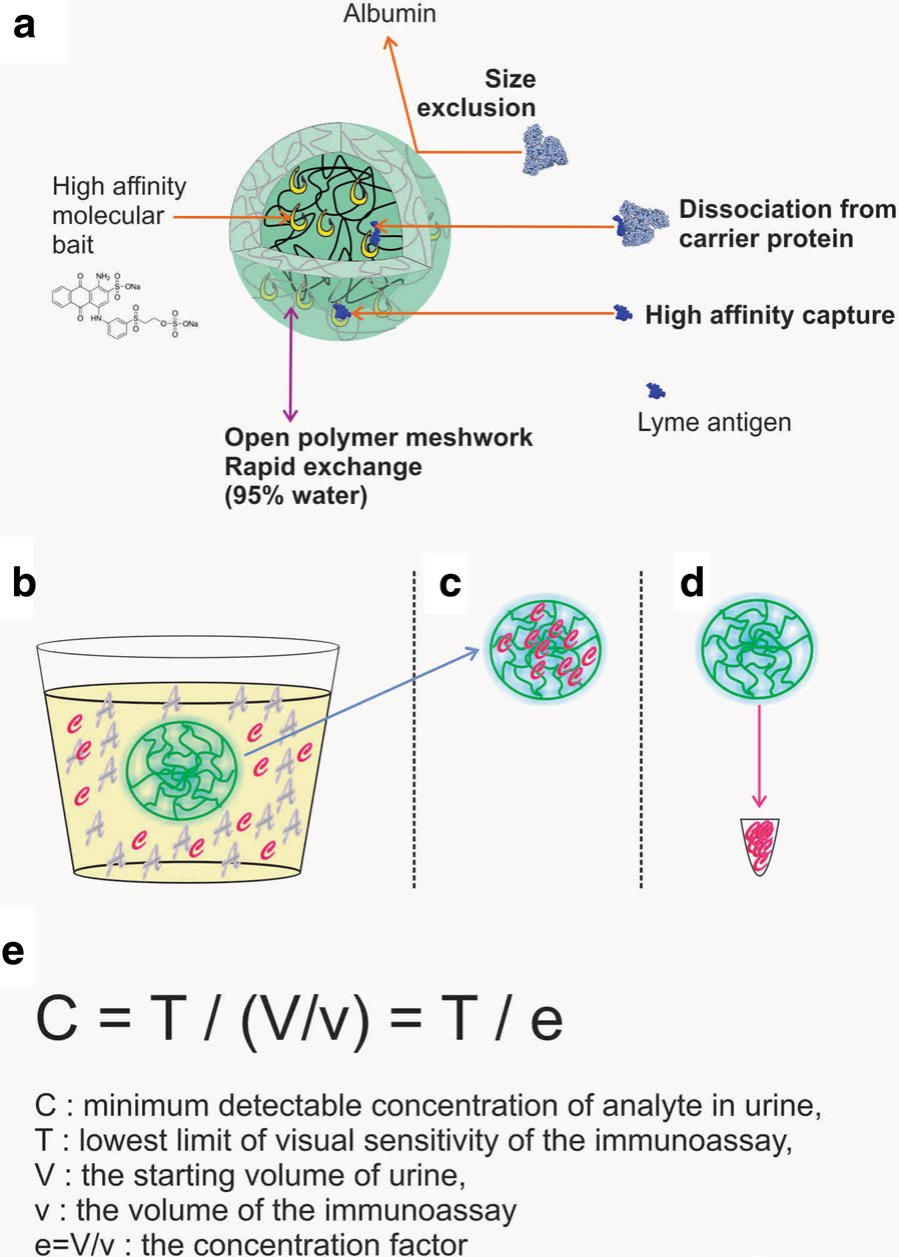
Nanotrap particles concentrate and preserve OspA and increase the effective sensitivity of the immunoassay. **a** Nanotrap particles are covalently functionalized with a high affinity bait that sequesters low abundance low molecular weight antigens. **b** Nanotrap particles are mixed with urine containing the Lyme antigen (*yellow* “*c*”) and vast excess of resident proteins (e.g. albumin). Nanotrap particles capture all the solution phase Lyme antigen (*red* “*C*”) and exclude high abundance resident proteins (*gray* “*A*”); **c** Nanotrap particles are separated from the urine. **d** Lyme antigens captured by the Nanotrap particles are eluted in a small volume (e.g. 0.015 mL). **e** Nanotrap particle pre-processing step increases the effective sensitivity of any analytical technology by a concentration factor e. Assuming the initial volume of urine is V = 40 mL and the final elution volume is v = 0.015 mL, the concentration factor e = V/v = 40/0.015 = 2,667

To overcome the previous physiologic and immunologic barriers to a sensitive and specific OspA immunoassay, we combined our novel nanotechnology to massively enhance the sensitivity of detection, with a mAb specific to a narrow C-terminal OspA domain. Our nanotechnology is Nanotrap particles containing a covalently anchored high affinity chemical bait that binds OspA antigen (Fig. 1a) [18, 19, 29, 30]. When the nanoparticles are introduced into urine, or any body fluid, they immediately capture and concentrate all the OspA in solution (Fig. 1b, c). The nanoparticle OspA cargo can then simply be eluted and measured by immunoassay (Fig. 1d). The amount of OspA captured is a function of the sample volume, since the Nanotrap particles bind virtually one hundred percent of the solution phase antigen in the entire volume (Fig. 1e) [18, 19, 29, 30]. We have previously shown that the high yield, preservation of captured antigen, sequestration of the antigen into a small volume, and exclusion of unwanted proteins, can dramatically improve immunoassay sensitivity more than one hundred fold [18, 19, 29, 30], without increasing the background. In this study, we used anti-OspA mAb clone 0551. We verified the anti-OspA mAb specificity by sequencing the OspA antigen mAb-binding epitope and verifying the epitope specificity by synthetic protein and peptide competition, and testing for cross-reactivity with non-*Borrelia* organisms. After validating the analytical sensitivity and specificity of our new OspA immunoassay, we then applied it to evaluate OspA shedding into the urine of 151 patients suspected of early stage, or recurrent, LB and 117 healthy controls (N = 268) (Table 1).

**Table 1:** Clinical features of patient enrolled in the diagnostic clinical trial

Patient characteristics	Number
Asymptomatic non-Lyme (healthy volunteers)	117
Symptomatic non-Lyme patients (non-Lyme patients in infectious disease clinic)	3
Untreated, clinical diagnosis of LB (positive diagnosis of Lyme disease with EM rash)	24
Antibiotic treatment for a clinical diagnosis of LB, Arthritis Pos, EM Neg (positive diagnosis of Lyme disease with arthritis symptoms)	6
EM rash present at the time of urine collection during the treatment course	10
Post-treatment with alleviation of symptoms (converted to EM Neg)	8
Patients being worked up for chronic or recurrent LB with negative or inconclusive serology	100

## Methods

### Ethics statement

Urine samples were collected under informed consent from patients suspected of having Lyme disease at any stage from four different community physician practices in Northern Virginia, a high risk geographic region for LB. This study met the requirements for IRB approval (Pro00008518, Chesapeake IRB). Matched coded clinical records and LB serology results were also provided under patient consent. Immunoassay testing for urinary OspA utilized Nanotrap particle pre-processing and western blotting to evaluate the OspA analyte molecular size, and OspA peptide competition was applied to fully confirm a positive test.

### Study design and human sample collection

Subjects with all stages of documented or suspected Lyme disease were considered eligible for the study. Mid-stream urine specimens were self-collected by 268 participants. Urine samples were stored at the collection sites (Care-ID, VA, USA; Frekko Primary Care, MD, USA; Internal medicine of Northern Virginia, VA, USA; Novant Health Virginia Internal Medicine & Primary Care, VA, USA) at −20 °C. Samples were frozen within 2 h of collection. Sample were then transferred to George Mason University and stored at −80 °C. Whole blood from each subject was drawn on the same day as urine collection. Each participant donated blood only once. The blood drawing procedure was performed at the physician’s office by a registered nurse. Six mL of whole blood was drawn into a red top tube. Blood samples were sent to Quest Diagnostics for Total Lyme Disease Antibody (EIA) and Lyme IgG and IgM Western Blot tests. Results of serological testing were recorded as well as treatment modalities and outcome, and used to correlate serology outcome with the presence of OspA detected in urine. Age matched, non-symptomatic patients with no history of Lyme disease as well as patients being evaluated for other non-Lyme infectious diseases were included in the control group. In addition, the study set contained Lyme borreliosis patients before and after antibiotic treatment.

### Epitope mapping of the anti-OspA mAb

In order to identify the epitope of the anti-OspA mAb, partial enzymatic digestion, western blotting and mass spectrometry analysis were performed using *Borreliaburgdorferi* (Bb) Lyme, Grade 2 Antigen (ARP American Research Products) and anti-OspA mAb clone 0551 (Santa Cruz). Bb (Lyme), Grade 2 Antigen was derived from Bb Strain B31 cultured in BSK II Medium. Microorganisms were harvested from growth medium and washed in PBS using low speed centrifugation. The pellet was resuspended in PBS and gently sonicated. The antigen preparation was evaluated by gel electrophoresis followed by silver staining and in gel trypsin digestion followed by mass spectrometry. In this study, partial enzymatic digestion was carried out as follows: 80 μL of Bb Lyme antigen Grade 2 (0.2 μg/ml) was acidified with 80 μL of 10 mM HCl (Fisher Scientific). 10 μL of 4 mg/mL pepsin solution (Sigma) was added to the sample and incubated for 1 h at RT. After the incubation, the sample was immediately purified with ZipTip (Millipore) according to manufacturer instructions and dried with Nitrogen evaporator (Microvap 118, Organomation Associate, Inc). The dried pepsinized sample was resuspended in 22.5 μL of water by repeated pipetting. An aliquot of 2.5 μL was subjected to western blot analysis with the anti OspA mAb described above in order to determine the smallest OspA pepsin fragment that retains antigenicity toward the antibody. The remaining amount of pepsinized Lyme antigen (20 μL) was subjected to SDS-PAGE fractionation and silver staining. Bands on the low molecular weight region of the gel that mirrored the signal on the western blot were cut using a razor blade and subjected to in gel digestion. Samples were reduced with 500 μL of 10 mM dithiothreitol (DTT, Fisher Scientific) in 50 mM ammonium bicarbonate (Fluka) and then alkylated with 500 μL of 50 mM iodoacetamide (Acros Organic) in 50 mM ammonium bicarbonate at room temperature in the dark for 20 min. Overnight enzymatic digestion was carried out with 0.5 µg sequencing grade trypsin (Promega) dissolved in 60 μL of 50 mM ammonium bicarbonate pH 8 at 37 °C. Solutions were separated from the gel pieces and saved in clean Eppendorf tubes. Aliquots of 60 μL of extraction buffer (50 % acetonitrile, 2 % acetic acid (Acros Organic) were added to the gel pieces and incubated at room temperature for 15 min. Solutions were separated from the gel pieces and combined with the previous ones. Samples were then dried using a nitrogen evaporator (Microvap 118, Organomation Associates). Samples were reconstituted in 6 µl of 0.1 % Formic Acid and analyzed with a Thermo LTQ Orbitrab Mass spectrometer. After sample injection by autosampler, the C18 column (0.2 × 50 mm, NanoLCMS Solutions) was washed for 2 min with mobile phase A (0.1 % formic acid), and peptides were eluted using a linear gradient of 0 % mobile phase B (0.1 % formic acid, 80 % acetonitrile) to 50 % mobile phase B in 90 min at 500 nL/min, then to 100 % mobile phase B for an additional 5 min. The LTQ mass spectrometer was operated in a data-dependent mode in which each full MS scan was followed by five MS/MS scans where the five most abundant molecular ions were dynamically selected for collision induced dissociation (CID) using a normalized collision energy of 35 %. Tandem mass spectra were searched against the *NCBI Borrelia burgdorferi* database with SEQUEST software using tryptic cleavage constraints. High-confidence peptide identifications were obtained by applying the following filter criteria to the search results: Xcorr versus charge 1.9, 2.2, 3.5 for 1+, 2+, 3+ ions; ΔCn > 0.1; probability of randomized identification e0.01. Fragments identified as OspA were used to design 7 different 20-mer peptides. Peptides mimicking the OspA fragments obtained with the procedure described above were synthesized by Peptide 2.0 Inc using standard solid phase techniques. Purity of peptides was assessed by chromatography and mass spectrometry and guaranteed to be higher than 98 %.

### Synthesis of dye functionalized, Nanotrap particles

Nanotrap particles were synthesized by precipitation polymerization and functionalized with organic reactive dyes through zero-link amidation reaction as previously described [19]. Briefly, 4.750 g of *N*-isopropylacrylamide (NIPAm) and 0.400 g of *N*, *N*′-methylenebisacrylamide (BIS) were dissolved in 500 mL of MilliQ water and filtered under vacuum into a three neck round bottom flask. 0.525 g of Acrylic Acid (AAc) were added, and the solution was purged with nitrogen for 30 min under medium stirring and then heated up to 70 °C. 0.276 g of Potassium Persulfate (Sigma Aldrich) was dissolved in 5 mL of H_2_O and added to the reaction in order to initiate the polymerization. The reaction was kept for 6 h at 70 °C. Particles were washed 5 times by centrifugation (19,000 rcf, 50 min, room temperature) with MilliQ H_2_O in order to eliminate unreacted monomer. Particles were resuspendend in a total volume of 600 mL of water. Particles functionalization was performed by condensation of the primary amine contained in the dye to the carboxylic group of the AAc present in the nanoparticles. Briefly, 40 mL of particles were centrifuged and the pellet was re-suspendend in 40 mL of 0.2 M NaH_2_PO_4_ pH 5. The particles were transferred into a round bottom flask and 2 mL of 1 % sodium dodecyl sulfate (SDS, Sigma) 1648 mg of N-(3 Dimethylaminopropyl)-*N*′-ethylcarbodiimide hydrochloride (EDC; Fluka Analytical) and 1224 mg of solid *N*-Hydroxysuccinimide (NHS; Sigma-ALBrich) were added. The reaction was kept for 15 min at room temperature under medium stirring, then the particles were centrifuged, and the pellet resuspended in 0.2 M Na_2_HPO_4_ pH 8. In parallel, 2 g of Remazol Brilliant Blue were dissolved in 100 mL of Na_2_HPO_4_ pH 8 and filtered twice with 0.2 µm nitrocellulose membrane disk filter (Millipore). Particles were then transferred to the dye containing solution and the reaction was held overnight at room temperature under medium stirring rate. The Nanotrap particles were washed by centrifugation (19,000 rcf, 50 min, room temperature) 6 times in order to eliminate the unreacted dye and then resuspended in a final volume of 40 mL with MilliQ H_2_O.

### Nanotrap particle characterization

Hydrodynamic diameter and temperature dependence of particle size were measured via photon correlation spectroscopy (Submicrometer Particle Size Analyzer, Beckam Coulter) using water as a diluent within a temperature gradient from 25 to 45 °C. Dye incorporation was measured weighing lyophilized Nanotrap particles before and after the coupling reaction.

### Urine sample handling prior to analysis

All urine used in this study was processed as follows. Urine samples were thawed in a water bath at room temperature. Urinalysis was performed on urine samples using Siemens Multistix 10SG. Specific gravity was measured with digital refractometer (Atago). Urine samples were then centrifuged at 3,700 rcf for 10 min at 25 °C to remove cellular debris. Supernatant was transferred in a new tube. Urine pH was measured and adjusted to 5.5 with 1 M HCl when necessary.

### Nanotrap particle performance assessment with model solutions

Bb Lyme antigen Grade 2 (American Research Products) spiked in human urine was used as model solution to test Nanotrap particle performance. OspA Lyme antigen (1.2 ng) was spiked in 40 mL of human urine collected from healthy volunteers and processed as described above and incubated with 1:10 Nanotrap particle suspension (5 mg/mL dry weight concentration)/urine solution volume. After 30 min incubation, Nanotrap particles were separated by centrifugation (16.1 rcf, 10 min, 25 °C). Two washes were performed by re-suspending the Nanotrap particles in 1 mL of MilliQ H_2_O and centrifuged at 16.1 rcf for 10 min at 25 °C. Nanotrap particles were incubated with 600 μL of elution buffer (70 % acetonitrile (Fisher Scientific), 10 % ammonium hydroxide (Sigma)) for 30 min at 25 °C. Samples were then centrifuged at 16,100 rcf for 15 min at 25 °C; the eluates were transferred to new tubes and 50 µL of a 50 mg/mL D-(+)-Trehalose dihydrate (Sigma) water solution was added. Eluates were then dried under nitrogen flow and analyzed by western blot.

### Patient urine processing with Nanotrap particles

In order to analyze patient urine samples, urine samples (40 mL) were transferred in Nalgene™ Oak Ridge High-Speed Polycarbonate Centrifuge Tubes and incubated with 4 mL of Nanotrap particles (5 mg/mL) for 30 min on a rocker at room temperature. Samples were then centrifuged at 19,000 rcf for 30 min at 25 °C. Two washes were performed by re-suspending the nanoparticles in 2 mL of MilliQ H_2_O and centrifuging at 16,100 rcf for 15 min at 25 °C. Nanotrap particles were incubated with 600 μL of elution buffer (70 % acetonitrile, 10 % ammonium hydroxide) for 30 min at room temperature. Samples were then centrifuged at 16,100 rcf for 15 min at 25 °C; the eluate was transferred to new tubes and 50 µL of D-(+)-Trehalose dihydrate solution (50 mg/mL in water) were added. Eluates were then dried under nitrogen flow and analyzed with western blot.

### OspA western blot analysis

Dried Nanotrap particle eluates were resuspended in 15 µL of MilliQ water by repeated pipetting. 5 µL of 4× sample buffer: 50 mM Tris HCl (Biorad) pH 6.8, 2 % SDS, 144 mM 2-mercaptoethanol (Fisher Scientific), 10 % glycerol (Sigma) and 0.01 % bromophenol blue (Fisher Scientific) was added and samples were heated at 100 °C for 10 min. Samples were loaded on 4–20 % Tris–Glycine gel (Invitrogen Corporation) and separated by SDS-PAGE gel electrophoresis. Gel was run in Tris–Glycine SDS running buffer using Novex X-Cell IITM Mini-Cell (Invitrogen Corporation) at 120 V for 90 min. Proteins were transferred onto a PVDF membrane (Millipore), blocked with a solution of 0.2 % I-Block (Applied Biosciences) and 0.1 % Tween 20 (Fisher) in PBS (Life Technologies). The membrane was incubated overnight with a mouse anti-OspA mAb (Santa Cruz, sc-58093, Clone ID 0551). This mouse mAb was purified from ascites fluid by protein A chromatography. The final preparation was formulated to a protein concentration of 100 μg/ml in 0.01 M phosphate buffered saline, pH 7.2 and contained 0.1 % sodium azide. The mAb was used at a 1:100 dilution in PBS supplemented with I-Block and Tween 20. After mAb incubation, the membrane was washed three times for 10 min with 0.2 % I-Block, 0.1 % Tween 20 in PBS. The membrane was incubated with a peroxidase conjugated goat anti-mouse IgG adsorbed against bovine, equine and human serum proteins (Sigma) diluted 1:5000 in 0.2 % I-Block, 0.1 % Tween 20 in PBS. Three washes of 10 min in 0.2 % I-Block, 0.1 % Tween 20 in PBS were performed. Proteins were detected with an enhanced chemiluminescence system (Supersignal West Dura, Thermo Fischer Scientific, cut off for detection: mid-femtogram levels of target proteins) on a Kodak MM4000 Imager [31].

### OspA dot blot analysis

Proteins and peptides (2 μL) were spotted with a capillary tube on a PVDF membrane previously wetted in methanol. The membrane was blocked with a solution of 0.2 % I-Block and 0.1 % Tween 20 in PBS. The membrane was incubated overnight with anti-OspA mAb clone 0551 diluted 1:100 in 0.2 % I-Block, 0.1 % Tween 20 in PBS (4 °C) and then washed three times for 10 min with 0.2 % I-Block, 0.1 % Tween 20 in PBS. The membrane was incubated with a peroxidase conjugated goat anti-mouse IgG adsorbed against bovine, equine and human serum proteins diluted 1:5,000 in 0.2 % I-Block, 0.1 % Tween 20 in PBS. Three washes of 10 min in 0.2 % I-Block, 0.1 % Tween 20 in PBS were performed. Proteins were detected with an enhanced chemiluminescence system (Supersignal West Dura, Thermo Fischer Scientific) on a Kodak MM4000 Imager.

### In solution competition assay and solid phase immunodepletion

In order to verify the specificity of band reactivity of the anti-OspA mAb clone 0551, a competition assay was developed. Prior to staining, the mAb was neutralized by incubation with a solution containing excess OspA or synthetic peptides containing partial OspA sequences. The mAb that was bound to the neutralizing protein or peptide was no longer available to bind to the epitope transferred on the western blot membrane. The blocked mAb and the mAb alone were used to probe duplicate western blots. All other parameters of the western blotting remained the same. The comparison of neutralized mAb to mAb alone showed which staining was specific: the specific staining was absent from the western blot membrane probed with the neutralized mAb. More in detail, 100 µL of anti-OspA antibody (0.1 mg/mL) was added to 900 µL of 0.2 % I-Block, 0.1 % Tween 20 in PBS and incubated overnight with 400 µL (0.1 mg/mL) of a custom made recombinant OspA (Genecopoeia). In parallel, Bb Lyme antigen Grade 2 (0.5 ng) was separated by 1-D gel electrophoresis and then transferred onto Immobilon PVDF membranes as previously described. OspA-saturated and un-modified antibodies were used to probe the PVDF membranes. Competition assays were also performed neutralizing the anti OspA mAb with Bb Lyme antigen Grade 2 (37 μg), 80 kDa OspA chimera recombinant protein (12 μg, Genway), and peptide fragments mimicking the antibody epitope (60 μg, Additional file 1: Table S1, Peptide 2.0).

The peptide OspA219-235 (Peptide 2.0) was utilized for solid phase affinity depletion of the mAb clone 0551. The peptide (300 μg) was deposited on ELISA plate wells. The wells were washed with PBS supplemented with 0.1 % Tween 20 and the excess peptide removed. The wells were then blocked with PBS supplemented with 0.2 % I-Block, 0.1 % Tween 20. The mAb clone 0551 (3 μg) was incubated with the solid phase adsorbed peptides overnight at 4 °C under rotation. After incubation, the supernatant was recovered and brought to a volume of 3 mL in PBS supplemented with 0.2 % I-Block and 0.1 % Tween 20. In parallel, 600 pg of Bb Lyme antigen Grade 2 were spiked in urine and processed through the Nanotrap particles. The immunodepleted mAb and the mAb alone were used side by side to stain membranes containing the Nanotrap particle eluates.

### Reproducibility and sensitivity of the urinary OspA Lyme assay

In order to assess intra-assay reproducibility of the urinary OspA Lyme assay, four experimental replicates were performed in one day as follows. Bb Lyme antigen Grade 2 (1.2 ng) was spiked in 40 mL of urine collected from healthy volunteers and incubated with 4 mL of Nanotrap particle suspension (5 mg/mL). Samples were processed as described above and the Nanotrap particle eluates were analyzed by western blot using anti-OspA mAb clone 0551.

In order to determine the lower limit of detection and the lower limit of quantitation of the urinary OspA assay, different quantities of Lyme antigen (1.2, 0.6, 0.3, 0.15, 0.075, 0.038, 0.019, and 0.009 ng) were spiked in 40 mL of human urine. Aliquots of 4 mL of Nanotrap particle suspension (5 mg/mL) were mixed with the urine samples and processed as described above. Nanotrap particle eluates were analyzed by Western blot. The experiment was repeated three times in three different days. Multiple batches of Nanotrap particles were used in this study. Batch validation and batch to batch reproducibility experiments were performed following the same protocol described above.

### Interfering substances and cross-reactivity with relevant non Bb infections

In order to exclude possible cross-reactivity of the mAb clone 0551 with interfering substances, a series of Nanotrap experiments were performed and analyzed using western blotting. Increasing amount of the following antigens were spiked in 40 mL of human urine in presence or absence of Bb Lyme antigen: bovine serum albumin, human healthy volunteer whole blood, *Bartonella henselae* lysate (ATCC 49793), *Babesia microti* (ATCC PRA-399), Epstein-Barr virus (EBV) Inactivated P3HR1 Cell Extract (Advanced Biotechnologies Inc.; 10-501-001), Herpes Simplex virus-1 (HSV-1) Inactivated Vero Cell Extract (Advanced Biotechnologies Inc; 10-515-001), Cytomegalovirus (CMV) HEK293 Cell Lysate (Sino Biological Inc.; 10202-VCCH1L), Hepatitis C Virus HEK293 Cell Lysate (Sino Biological Inc.; 10202-VCCH1L). These samples were processed following the protocol described above.

### Data analysis

Statistical Chi squared test for equality of proportions was applied in order to correlate urinary OspA outcome (detectable or non-detectable) to clinical LB diagnosis and serology. Power calculations were performed in order to estimate the power of the test, given the number of samples in each group, the proportions of urinary OspA outcome, and a significance level of 0.05. Calculations were performed using R software (http://www.r-project.com).

Western blotting band intensity was quantified with ImageJ software (http://imagej.nih.gov/ij/index.html) by selecting the area of interest and calculating area, mean and standard deviation of selection per software instructions. Blast analysis and protein alignment was performed using pBLAST [32]. Search parameters were as follows: query sequence: KTSTLTISVNSKKTTQLVFTKQDTITVQKYDSAGT, Database Name: non redundant, Program: BLASTP 2.2.31+.

## Results

### Mass spectrometry sequencing and peptide competition reveals the OspA C-terminal epitope recognized by the anti-OspA mAb

**Fig. 2:**
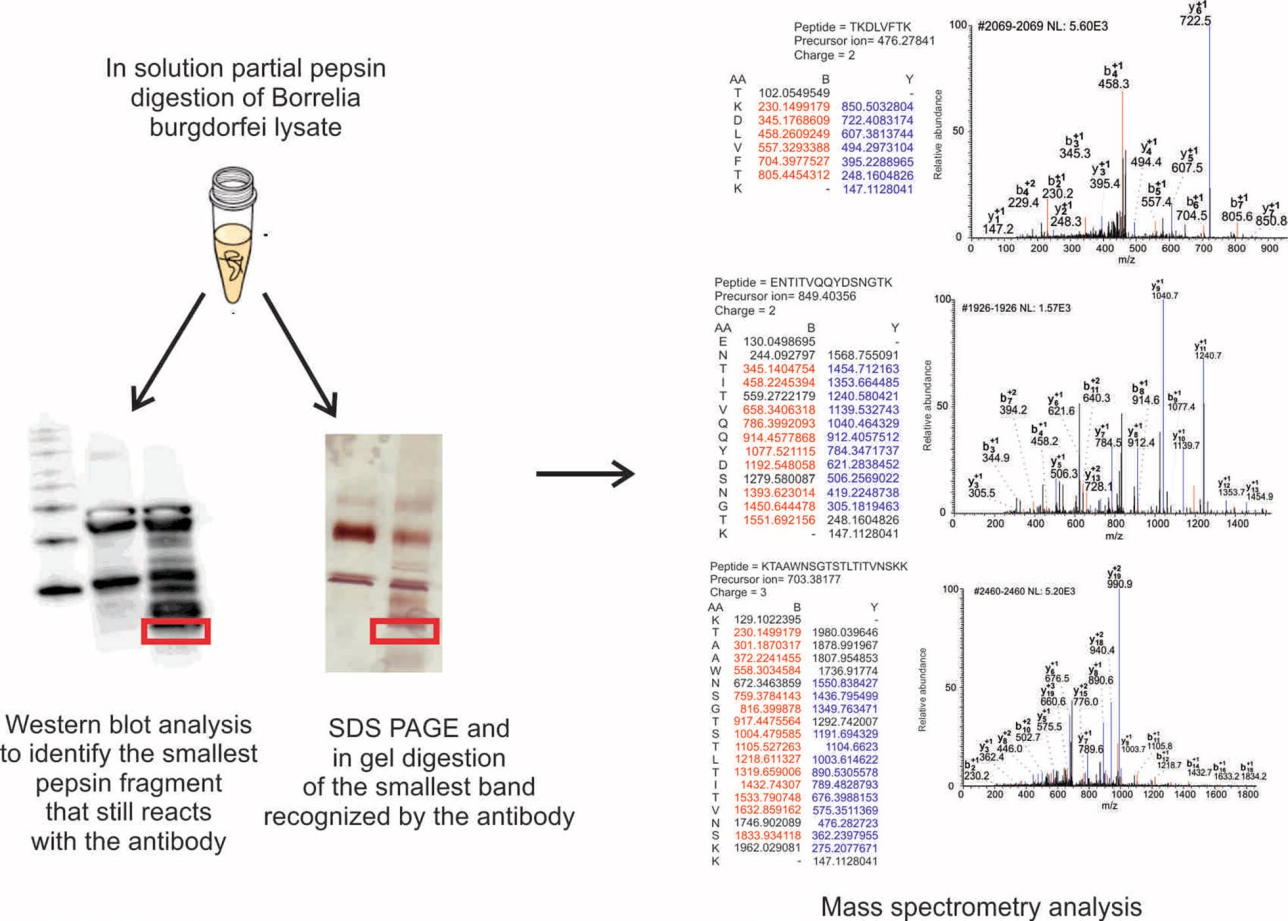
Mapping the OspA antigen epitope. Bb Lyme antigen was partially digested with pepsin and the protease derived fragments were split into two aliquots. One aliquot was analyzed by western blotting with the anti-OspA mAb clone 0551. In parallel, the second aliquot of pepsin fragments was analyzed by SDS PAGE and silver stained. The band in the SDS PAGE mirroring the smallest peptide fragment recognized by the mAb was cut out and processed for mass spectrometry analysis. MS/MS spectra of the smallest peptides that reacted with the mAb are shown

**Fig. 3:**
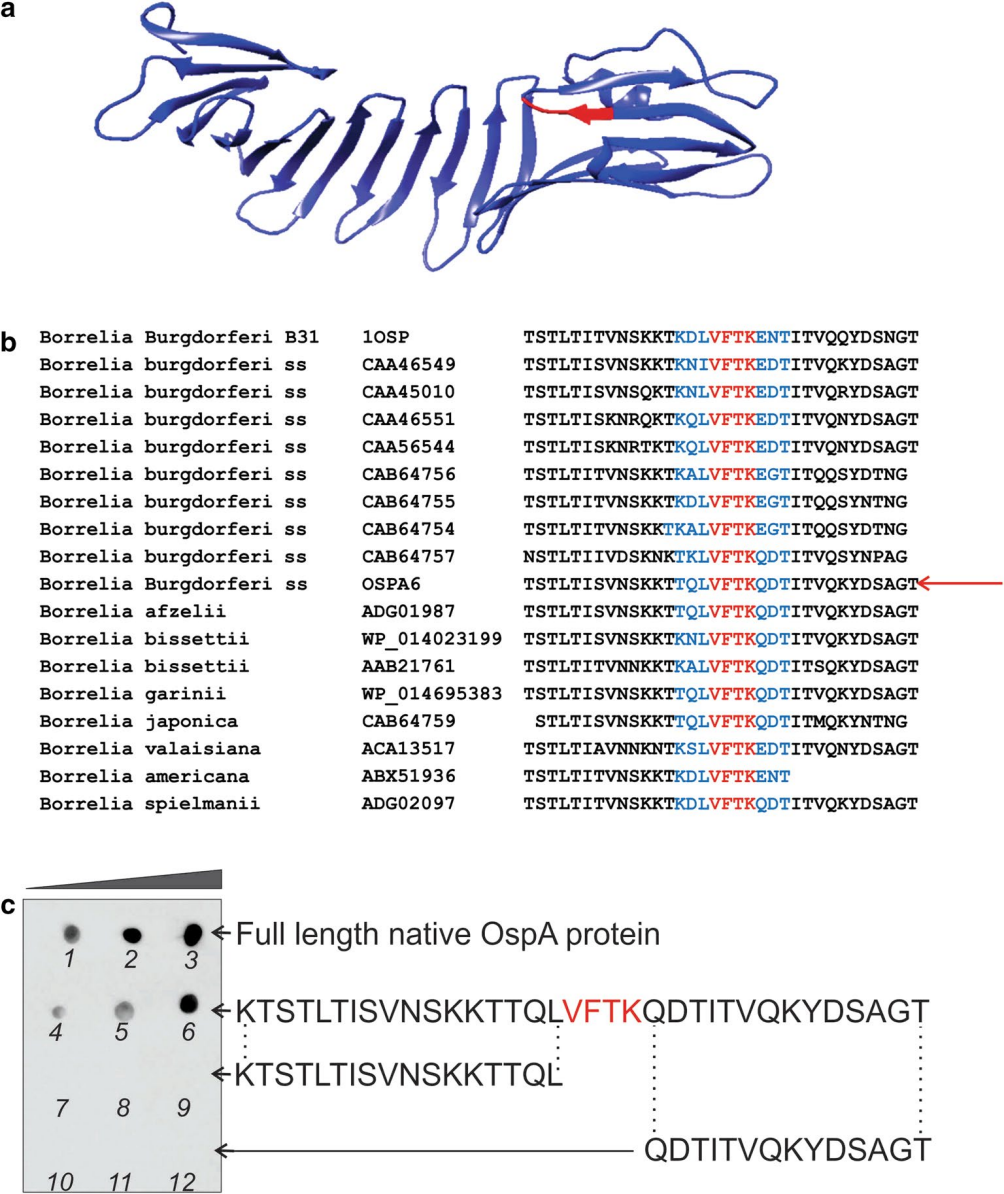
Narrow OspA236-239 region is conserved across different *Borrelia* species and binds to mAb clone 0551. **a** Crystallography structure of OspA (Protein Data Bank PDB ID# 1FJ1): the epitope of the mAb is highlighted in *red*. **b** BLAST search against different *Borrelia* strains and species shows that the mAb clone 0551 epitope is highly conserved whereas the flanking regions are variable. **c** Synthetic peptides mimicking the OspA236-239 region interact with the mAb in a dose dependent manner (*dot* blot analysis, *1*, *2*, *3* = Bb Lyme antigen 0.5, 5, and 10 ng, respectively; *4*, *5*, and *6* = OspA219-253 0.5, 1, and 2 μg, respectively; *7*, *8*, and *9* = OspA219-235 0.5, 1, and 2 μg, respectively; *10*, *11*, and *12* = OspA240-253 0.5, 1, and 2 μg, respectively). Negative control peptides (OspA219-235 and OspA240-253) containing flanking regions but lacking the OspA236-239 sequence were devoid of immunoreactivity with the mAb clone 0551

**Fig. 4:**
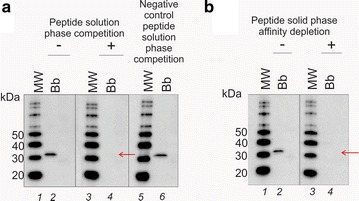
Peptides containing the narrow OspA236-239 region were successfully utilized for antibody competition and immunodepletion. **a** 600 pg of Bb Lyme antigen Grade 2 were spiked in human urine. Samples were processed through the Nanotrap particles and analyzed by western blot. *Lane 2, 4* and *6* were obtained staining the western blot membranes with the mAb clone 0551 alone, the mAb neutralized with OspA219-253 peptide, and the mAb neutralized with a combination of OspA219-235 and OspA240-253, respectively. The peptide containing the OspA236-239 region successfully competed the mAb, whereas peptide missing the OspA236-239 region failed to compete the mAb clone ID 0551. **b** Peptide OspA219-235 was utilized for solid phase affinity depletion of the mAb clone 0551. The peptide (30 μg) was deposited on ELISA plate wells. The wells were washed and the excess peptide removed. The wells were blocked with PBS supplemented with 0.2 % I-Block, 0.1 % Tween 20. The mAb clone 0551 (3 μg) was incubated with the solid phase adsorbed peptides overnight. After incubation, the supernatant was recovered and brought to a volume of 3 mL in PBS supplemented with 0.2 % I-Block and 0.1 % Tween 20. In parallel, 600 pg of Bb Lyme antigen Grade 2 were spiked in urine and processed through the Nanotrap particles (*lane* *2* and *4*). *Lane* *2* and *4* were obtained staining the western blot membranes with the mAb alone (3 μg) and the mAb after immunodepletion, respectively. There is no immunoreactivity in the mAb preparation after immunodepletion (*lane* *4*). This is a further confirmation of the absence of non-specific signal in the mAb clone 0551 preparation

The epitope of OspA recognized by the mAb clone 0551 was sequenced and verified by peptide dot blot and peptide competition. First, the Bb Lyme antigen Grade 2 (81 % OspA content, Additional file 1: Figure S1) was partially digested with pepsin and the protease derived fragments were split into two aliquots. One aliquot was analyzed by western blotting with the anti-OspA mAb clone 0551. In parallel, the second aliquot of pepsin fragments was analyzed by SDS-PAGE and silver stained. The band in the SDS-PAGE mirroring the smallest peptide fragment recognized by the mAb was cut out and processed for mass spectrometry analysis (Fig. 2). The fragment KTSTLTISVNSKKTTQLVFTKQDTITVQKYDSAGT (OspA219-253, Fig. 2, Additional file 1: Table S1) was the smallest sequence that reacted with the mAb clone 0551. This sequence is located on the C-terminal region of the protein (Fig. 3a, b). Two overlapping peptides, Peptide 5 (OspA219-239) = KTSTLTISVNSKKTTQLVFTK and peptide 6 (OspA234-253) = QLVFTKQDTITVQKYDSAGT (Additional file 1: Table S1), were synthesized and evaluated against control peptides from other regions of the molecule for their reactivity with the mAb (Fig. 3c, Additional file 1: Figure S2). The overlapping region of these peptides (OspA236-239 = VFTK) was found to be necessary and sufficient for antibody recognition via dot blot (Additional file 1: Table S1, Fig. 3c), solution phase competition and immunoaffinity solid phase competition (Fig. 4). The flanking regions, highly variable in the *Borrelia burgdorferi* sensu *strictu* and across different *Borrelia* species were devoid of immunoreactivity with the mAb clone 0551 (Figs. 3, 4, Additional file 1: Table S1).

### The anti-OspA mAb epitope is conserved in common pathogenic species of *Borrelia*

The antigenic epitope OspA236-239 is highly conserved among major pathogenic *Borrelia* strains, which include: *Borreliaburgdorferi* sensu stricto, several *Borreliaburgdorferi* sensu lato (*Borreliagarinii*, *Borreliavalaisiana*, *Borreliabissettii*, *Borreliaafzelii*, and *Borreliaspielmanii*) and additional more recently characterized pathogenic species (Additional file 1: Table S2). BLAST analysis [32] performed on the sequence of the fragment OspA219-253 KTSTLTISVNSKKTTQLVFTKQDTITVQKYDSAGT identified by mass spectrometry showed that it was not homologous to any human protein and not homologous to any other non-*Borrelia* spirochetes (Additional file 1: Table S2).

### Nanotrap particles functionalized with a high affinity chemical bait capture urine OspA with high efficiency and yield

**Fig. 5:**
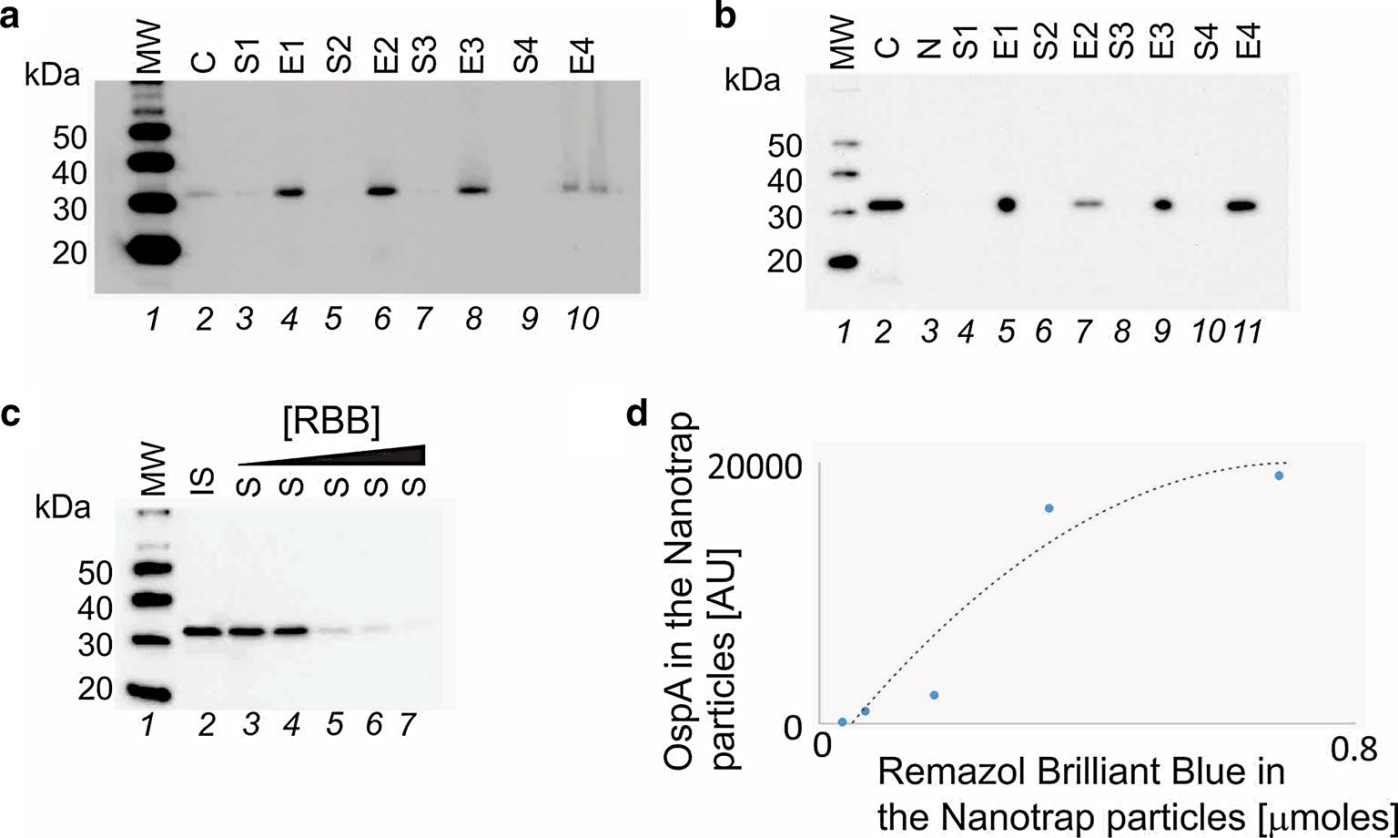
Remazol Brilliant Blue Nanotrap particles show the highest affinity for OspA among the tested dyes. **a** Nanotrap particles Lyme antigen (1 ng) was spiked in 500 µL of human urine from healthy volunteers and incubated with Nanotrap particles functionalized with different dyes. After Nanotrap particles processing, Lyme antigen is successfully depleted from supernatants (S) and easily detectable in the eluate (E). *Lanes* *1* ladder; *2* Lyme antigen 0.1 ng; *3* Supernatant Remazol Brilliant Blue Nanotrap 1; *4* Eluate Remazol Brilliant Blue Nanotrap 1; *5* Supernatant Remazol Brilliant Blue Nanotrap 2; *6* Eluate Remazol Brilliant Blue Nanotrap 2; *7* Supernatant Reactive Blue 4 Nanotrap; *8* Eluate Reactive Blue 4 Nanotrap; *9* Supernatant Diamine Green Nanotrap; *10* Eluate Diamine Green Nanotrap. **b** Nanotrap particles without dye bait can volume sequester some antigen but not concentrate the analyte from the surrounding solvent volume, in absence of affinity capture. The amount of OspA in the dye free particles is approximately 10 % of the control solution reflecting the urine solution/Nanotrap particle volumetric ratio (10:1). Lanes *1* ladder; *2* Lyme antigen 1 ng; *3* Negative control = urine without Bb antigen, *4* Supernatant vinyl phenyl boronic acid Nanotrap 1; *5* Eluate vinyl phenyl boronic acid Nanotrap 2; *6* Supernatant acrylic acid Nanotrap; *7* Eluate acrylic acid Nanotrap; *8* Supernatant allylamine Nanotrap; *9* Eluate allylamine Nanotrap; *10* Supernatant Remazol Brilliant Blue Nanotrap; and *11* Eluate Remazol Brilliant Blue Nanotrap. **c** Relationship between the Remazol Brilliant Blue in the particles and depletion of the antigen in the supernatant: 100 % antigen depletion from the solution phase and saturation of binding. *Lane* *1* ladder, *lane* *2* initial solution, *lanes* *3*–*7* supernatants after incubation of a urine solution containing 0.6 ng of Bb antigen with Nanotrap particles functionalized with increasing concentration of Remazol Briliant blue: 34, 68, 171, 343, 686 n moles, respectively. **d** ImageJ quantification of the optical density in (**c**). Y axis: Antigen sequestered in the Nanotrap particles obtained as difference between the initial solution (*lane* 2) and the supernatants (*lanes* 3–7) in (**c**). X axis: μ moles of Remazol Brilliant blue in the Nanotrap particles

**Fig. 6:**
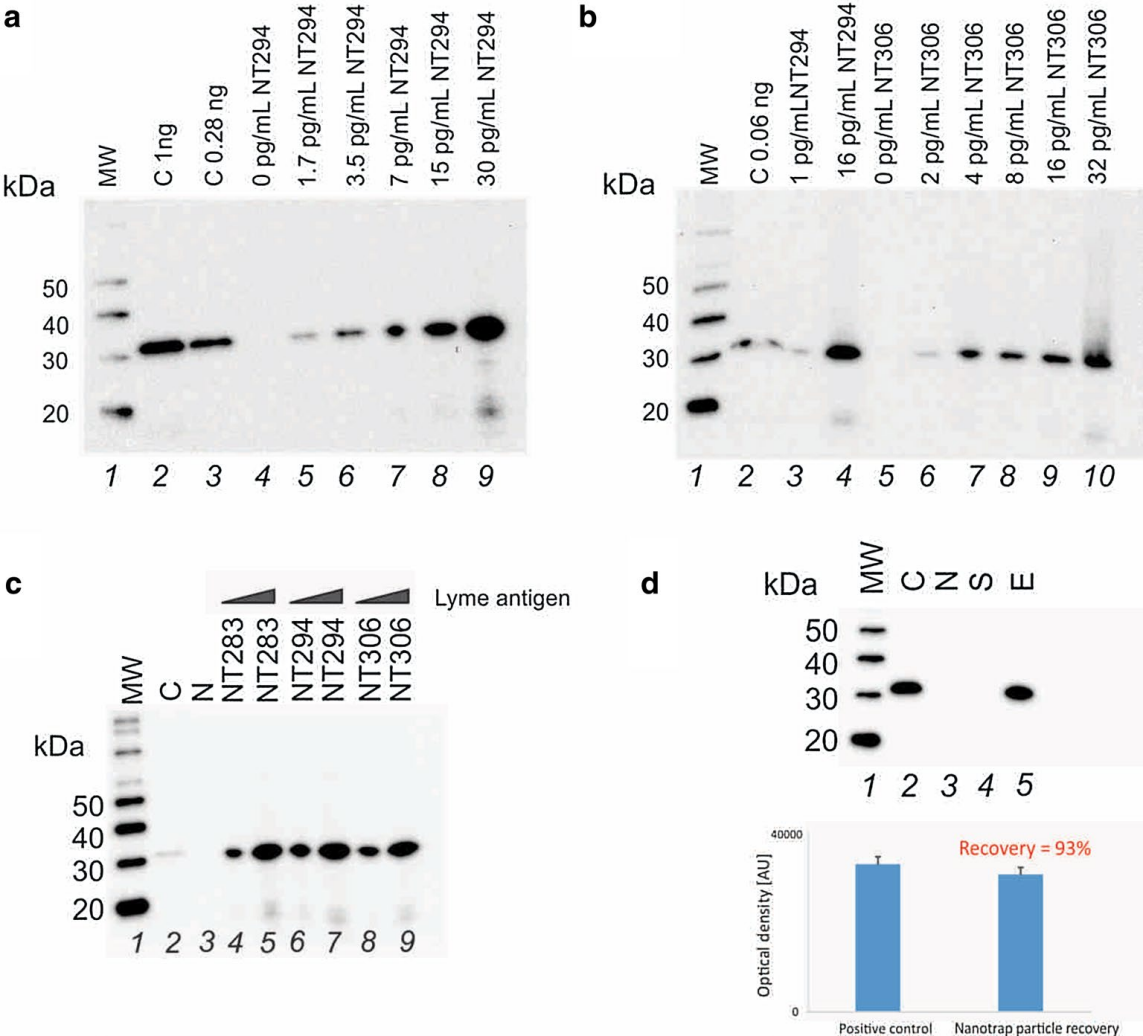
Nanotrap particle batch validation and batch to batch reproducibility. **a** Nanotrap particles are incubated with increasing amount of Bb Lysate spiked in 40 mL of urine; *Lane* *1*: Ladder; *Lane* *2*: Positive control Bb Lyme antigen Grade 2 1 ng; *Lane* *3*: Positive control Bb Lyme antigen Grade 2 0.28 ng; *Lanes* *4–9*: eluate of Nanotrap particles incubated with increasing concentrations of Bb antigen in 40 mL of urine: 0; 1.7; 3.5; 7; 15; 30 pg/mL, respectively **b** Performance comparison of two batches of Nanotrap particles, NT294 and NT306. *Lane 1*: Ladder; *Lane 2*: Positive control Bb Lyme antigen Grade 2 60 pg; *Lane 3*: eluate of Nanotrap particles batch NT296 incubated with 1 pg/mL Bb antigen urine solution (40 mL); *Lane 4*: eluate of Nanotrap particles batch NT296 incubated with 16 pg/mL Bb antigen urine solution (40 mL); *Lane 5*–*10*: eluate of Nanotrap particles batch NT306 incubated with increasing concentrations of Bb antigen urine solution (40 mL) 0; 2; 4; 8; 16; 32, respectively. **c** Performance comparison of multiple batches of Nanotrap particles (%CV = 9 % and 5 % at 2 pg/mL and 16 pg/mL Bb antigen in 40 mL of urine, respectively). Lane 1: ladder; lane 2: Positive control Bb Lyme antigen Grade 2 16 pg; lane 3: eluate of Nanotrap particles batch NT283 incubated with 40 mL of urine without Bb Lyme antigen Grade 2; *lanes* 4–5: eluates of Nanotrap particles batch NT283 incubated with 2 and 16 pg/mL Bb Lyme antigen Grade 2 urine solution (40 mL); *lanes* 6–7: eluates of Nanotrap particles batch NT294 incubated with 2 and 16 pg/mL Bb Lyme antigen Grade 2 urine solution (40 mL); *lanes* 8–9: eluates of Nanotrap particles batch NT306 incubated with 2 and 16 pg/mL Bb Lyme antigen Grade 2 urine solution (40 mL). **d** Yield of Nanotrap particle pre-processing is 93 %. *Lane 1*: ladder; *lane 2*: Bb antigen 320 pg, lane 3: eluate of Nanotrap particles batch NT283 incubated with 40 mL of urine without Bb Lyme antigen; *lane 4*: supernatant; *lane 5*: eluate of Nanotrap particles batch NT283 incubated with 8 pg/mL Bb Lyme antigen Grade 2 urine solution (40 mL)

Nanotrap particles were functionalized with a series of chemical baits [18, 19] that bind solution phase analytes with high affinity (Fig. 5, Additional file 1: Table S3 and S4, Figure S3). The reactive dye Remazol Brilliant Blue [19] had the highest affinity for OspA in urine (Fig. 5a, b). In addition, we document the relationship between the amount of dye bait in the particles and the depletion of antigen from the supernatant, providing clear evidence of 100 % antigen depletion from the solution phase and saturation of binding (Fig. 5c, d). The Nanotrap particles with no dye bait can volume sequester some antigen [18, 19], but not concentrate the analyte from the surrounding solvent volume, because there is no affinity capture without the dye bait [19] (Fig. 5b). Multiple Nanotrap particle batches were used throughout the study. Under CAP laboratory certification, we conducted routine batch to batch verification, and batch validation, with specific release criteria (%CV < 10 %) (Fig. 6). A series of v/v ratios (nanoparticle volume/sample volume) were tested in order to optimize OspA capture (achieving greater than 95 % capture and elution yield of all solution phase OspA (Additional file 1: Fig. S4). Nanotrap particle pre-processing will increase the effective analyte concentration more than 2000 fold, as a function of the volumetric ratio (Fig. 1e) (The initial volume of urine is V = 40 mL and the final elution volume is v = 0.015 mL, V/v = 40/0.015 = 2667).

### Quantitative OspA C-terminal peptide western blotting following Nanotrap particle processing of urine yields high precision and high sensitivity

**Fig. 7:**
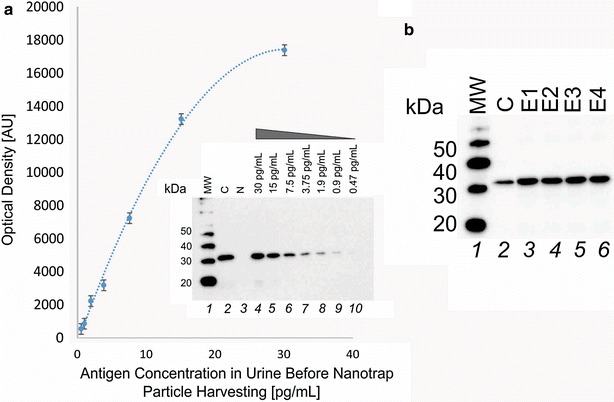
Lower limit of detection/quantitation and reproducibility (%CV = 7 %) of the urinary OspA Lyme test. **a** Sensitivity studies on three independent replicates: lower limit of detection (LLD) is 1.7 pg/mL. The lower limit of quantitation (LLQ) is 4.2 pg/mL for a 40 mL urine sample input volume. (Background estimate = 1071 AU, standard deviation (SD) = 323 AU. LLD = background + 2*SD, LLQ = background + 10× SD; polynomial equation y = −19.026x^2^ + 1160x − 248.76, R^2^ = 0.9971, was used to estimate the corresponding x value (1.7 pg/mL and 4.2 pg/mL, respectively)). Insert: *1* ladder; *2* Bb Lyme antigen control 1000 pg; *3* Eluate from Nanotrap particles incubated with 40 mL of volunteer urine containing no Bb Lyme antigen, negative control; *4*–*10* Eluate from Nanotrap particles incubated with 40 mL of volunteer urine containing decreasing concentrations of OspA, 30, 15, 7.5, 3.75, 1.9, 0.9, and 0.47 pg/mL, respectively. **b** Within run assay %CV is 7 %. Lyme antigen (1200 pg) was spiked in 4 urine aliquots (40 mL) and incubated with 4 mL of Nanotrap particles. Band intensity was measured with Image J. Within run %CV = 7 %. *1* Ladder; *2* OspA Lyme antigen control 200 pg, *3*–*6* Replicates of Nanotrap particle processed spike-in samples

Spiked-in samples were pre-processed with Nanotrap particles and western blot immunoassay followed by densitometric scanning. Densitometric analysis of the OspA specific band indicated a high level of intra-assay precision (n = 4, %CV = 7 %) for the nanoparticle concentration/immunoblotting method achieving a lowest limit of detection (LLD) of 1.7 pg/mL starting from 40 mL of urine (Fig. 7). The dynamic range is 1.7–30 pg/mL.

### Lack of cross-reactivity with relevant non *Borrelia* infections

**Fig. 8:**
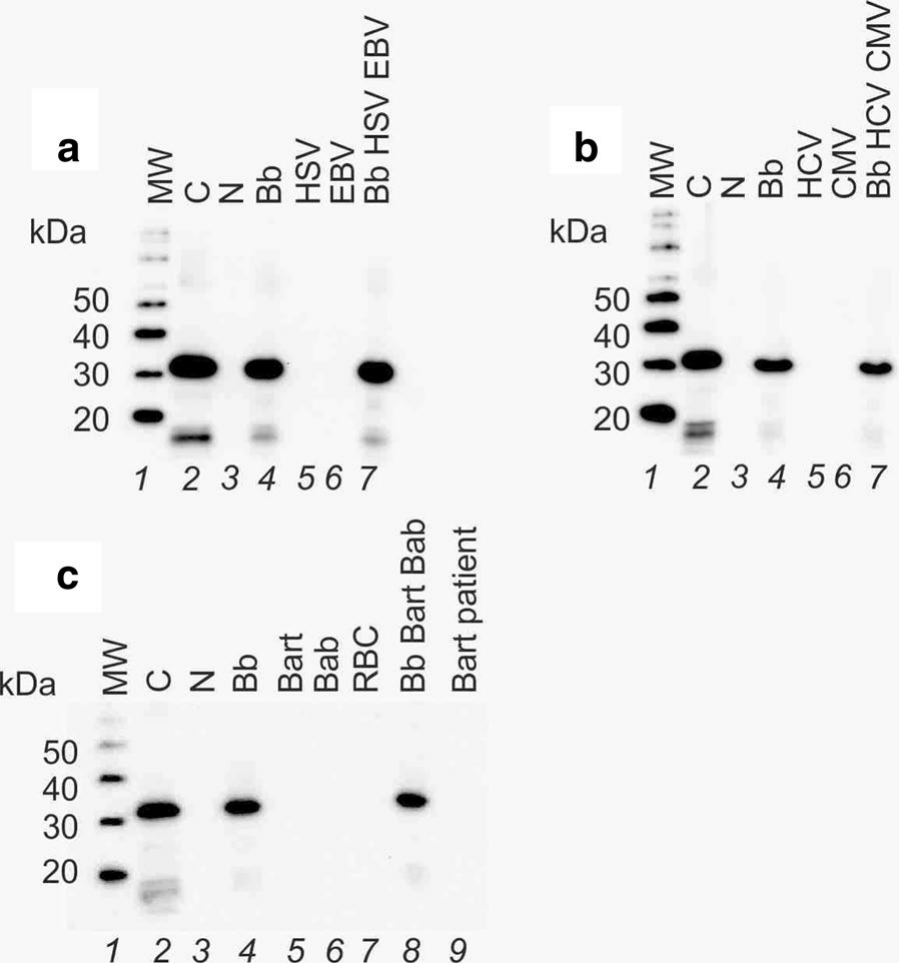
Viral and Lyme disease co-infection pathogens were devoid of immunoreactivity with the mAb clone 0551. Infection with these common non-Lyme pathogens do not generate a false positive for *Borrelia* in the present Nanotrap test. **a** Herpes simplex 1 and Epstein Barr viral lysates were mixed with urine in presence and absence of Bb antigen. Samples were processed with Nanotrap particles and analyzed with western blot. *Lane 1* ladder, *lane 2* Bb antigen 1 ng; *lanes 3–7* eluates of Nanotrap particles incubated with urine without Bb antigens (*lane 3*), 25 pg/mL Bb antigen in urine 40 mL (*lane 4*), HSV-1 lysate (1 μg in 40 mL of urine) (*lane 5*), EBV lysate (1 μg in 40 mL of urine) (*lane 6*), HSV-1 lysate (1 μg in 40 mL of urine) plus EBV lysate (1 μg in 40 mL of urine) plus Bb antigen (1 ng in 40 mL of urine) (*lane 7*). (**B**) Hepatitis C and Cytomegalovirus viral lysates were mixed with human urine in presence and absence of Bb antigen. *Lane 1*: ladder, *lane 2* Bb antigen 1 ng; *lanes 3*–*7*: eluates of Nanotrap particles incubated with urine without Bb antigen (*lane 3*), 25 pg/mL Bb antigen in urine 40 mL (*lane 4*), HCV lysate (1 μg in 40 mL of urine) (*lane 5*), CMV lysate (1 μg in 40 mL of urine) (*lane 6*), HCV lysate (1 μg in 40 mL of urine) plus CMV lysate (1 μg in 40 mL of urine) plus Bb antigen (1 ng in 40 mL of urine) (lane 7). **c** *Bartonella henselae* and *Babesia microti* lysates were spiked in human urine in presence and absence of Bb antigen. *Lane 1*: ladder, *lane 2*: Bb antigen 1 ng; *lanes 3–9*: eluates of Nanotrap particles incubated with urine without Bb antigen (*lane 3*), 25 pg/mL Bb antigen in urine 40 mL (*lane 4*), *Bartonella* lysate (1 ng in 40 mL of urine) (*lane 5*), *Babesia* lysate (10 ng in 40 mL of urine) (*lane 6*), red blood cells (10 ng in 40 mL of urine) (*lane 7*), *Bartonella* lysate (10 ng in 40 mL of urine) plus *Babesia* lysate (10 ng in 40 mL of urine) plus Bb antigen (1 ng in 40 mL of urine) (*lane 8*), 40 mL of urine of a patients with *Bartonella* positive and *Borrelia* negative serology at the time of urine collection (*lane 9*)

OspA antigen, at the expected full length size of the molecule, was the single band detected by Western blotting after Nanotrap processing of OspA containing human urine spiked with viral lysates (Herpes Simplex Virus-1, Epstein-Barr virus, Cytomegalovirus, and Hepatitis C Virus), and bacterial lysates (Lyme disease co-infection microorganisms *Bartonella henselae* and *Babesia microti*) (Fig. 8). Thus viral and non *Borrelia* bacterial lysates do not interfere with the Nanotrap particle capture of the antigen and with the mAb recognition and are devoid of immunoreactivity with the mAb. Similarly, increasing concentrations of interfering Bovine Serum Albumin and blood (hemoglobin) showed no cross-reactivity with the mAb (Additional file 1: Figure S5). Western blotting of urine samples from a positive control LB urine verified that Nanotrap particle pre-processing step is necessary to detect the expected low concentration of OspA in urine of patients with LB (Additional file 1: Figure S6).

### Positive cases were confirmed by competition with OspA protein

**Fig. 9:**
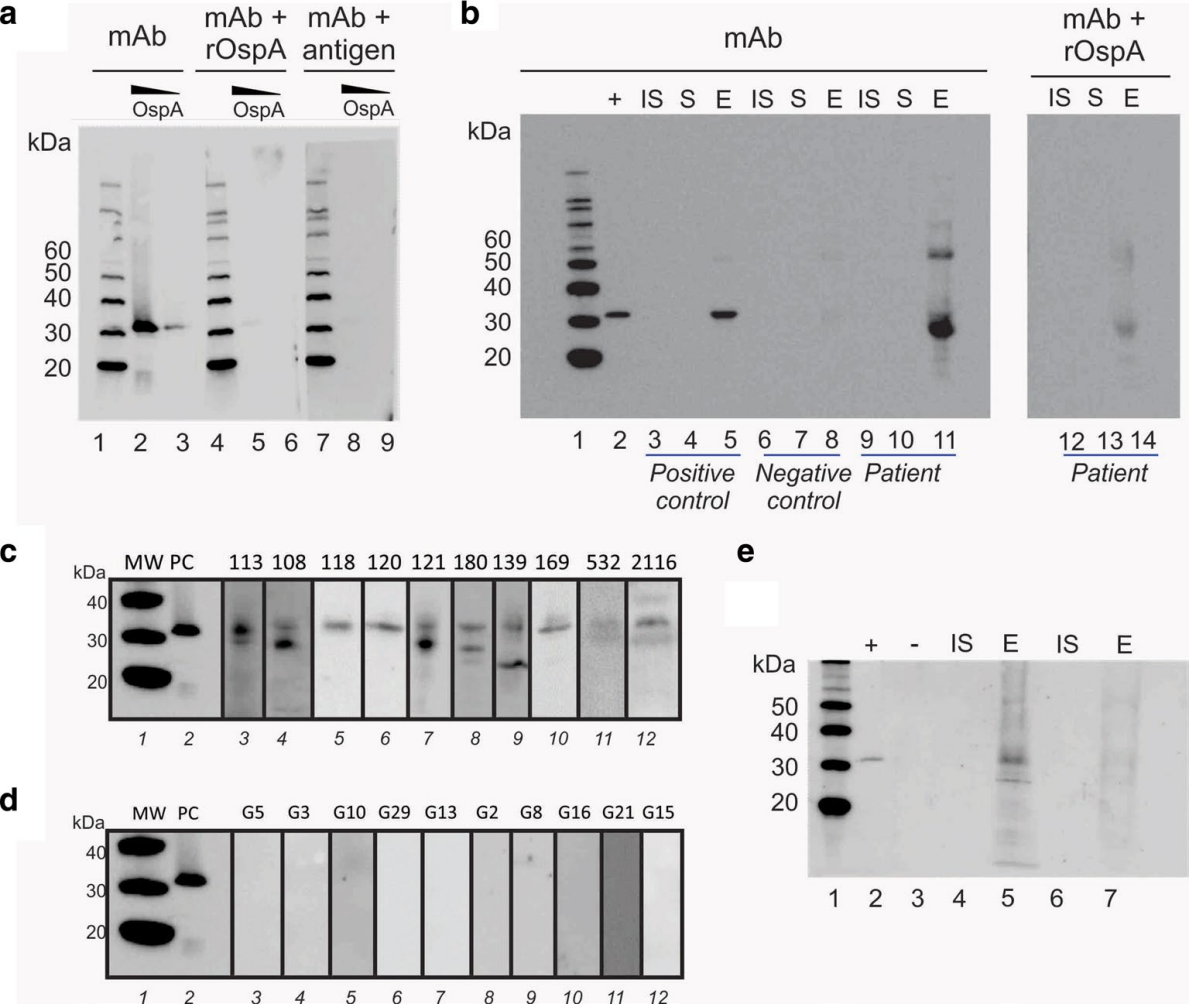
Nanotrap particles are necessary to detect urinary OspA in the urine of acute Lyme patients. Urinary OspA bands reverts to undetectable after successful treatment. Band specificity is assessed through competition assay. **a** Conditions for an optimal competition assay were established. 1 ng (*lanes 2*, *5*, and *8*) and 0.05 ng (lane 3, 6, and 9) of Bb Lyme antigen Grade 2 were spiked in urine. *Lanes 2* and *3* were obtained with mAb clone 0551 alone. *Lanes 5* and *6* were obtained with mAb neutralized with recombinant OspA. Reduced signal demonstrates high specificity. Lanes 8 and 9 were obtained with the mAb neutralized with Bb Lyme antigen Grade 2. Absence of signal with the neutralized antibody demonstrates specificity to OspA. **b** Competition assay was performed on all patient samples. Positive test outcomes show minimal or no binding of neutralized mAb to specific bands. Example competition assay for a Lyme disease patient is shown. *1* ladder; *2* Bb Lyme antigen Grade 2 OspA 1 ng; *3* initial Solution positive control (0.5 ng in 40 mL of urine processed through the Nanotrap particles); *4* supernatant positive control (0.5 ng in 40 mL of urine processed through the Nanotrap particles); *5* eluate positive control (0.5 ng in 40 mL of urine processed through the Nanotrap particles); *6* initial solution negative control (40 mL of urine processed through the Nanotrap particles); *7* supernatant negative control (40 mL of urine processed through the Nanotrap particles); *8* eluate negative control (40 mL of urine processed through the Nanotrap particles); *9* initial solution patient 180; *10* supernatant patient 180; *11* Eluate patient 180; *12* initial solution patient 180; *13* supernatant patient 180; *14* eluate patient 180. Membrane containing *Lanes 1–11* was probed with the mAb alone, whereas membrane containing *lanes 12–14* was probed with the mAb clone 0551 neutralized with recombinant OspA. **c** *Lane 1* ladder, *lane 2* Bb antigen 1 ng; *Lanes 3*–*12*: example of patient urine samples demonstrating presence of OspA. Positive OspA bands are normally visible in the 28–30 kDa range although lower molecular bands can be detected and successfully competed suggesting the presence of smaller-than-full-lenght OspA C-terminal domain containing protein fragments in urine. **d** Nanotrap antigen test results on a representative sub-group of the 117 healthy volunteers (Table 1). *Lane 1* ladder, *lane 2* Bb antigen 1 ng; *Lanes 3*–*12* example of patient urine samples demonstrating absence of OspA. **e** The OspA band is not detectable in the urine of acute stage Lyme patients after successful treatment. *Lane 1* ladder; *lane 2* Bb Lyme antigen Grade 2 in urine 0.1 ng; *lane 3* negative control urine with no OspA; lane 4 Initial solution (=urine without Nanotrap particle pre-processing) of patient 120 before treatment; *lane 5* eluate of patient 120 before treatment; lane 6 Initial solution patient 120 after treatment; *lane 7* Eluate patient 120 after treatment

Protein competition using a recombinant protein containing the OspA C-terminal mAb binding domain OspA236-239 was employed for the evaluation of patient urine specimens to ensure complete immunoassay specificity and to evaluate the presence of C-terminal fragments of OspA (Fig. 9a). A direct Western immunoblot (Fig. 9b) and a competition assay were performed on the patient’s urine sample (Fig. 9b). An individual patient’s urinary OspA was scored as positive only if both assay methods were judged positive. Positive OspA bands are normally visible in the 28–30 kDa range although lower molecular bands can be detected and successfully competed suggesting the presence of smaller OspA C-terminal domain protein fragments in urine. In some patients a high molecular weight band at ~60 kDa was detected and this was determined to be a dimer of OspA by mass spectrometry sequencing (Additional file 1: Figure S1). The higher molecular weight band was fully competable by the recombinant OspA (Fig. 9b).

### *Borrelia* OspA is shed in the urine of early stage Lyme patients

#### Untreated patients with a clinical diagnosis of acute LB

Urinary *Borrelia* OspA protein was present in untreated patients who were suspected of having early stage cutaneous LB, objectively classified under CDC guidelines by the presence of a characteristic erythema migrans (EM) rash, with or without concurrent symptoms such as fever, malaise, or neurologic symptoms (e.g. Bell’s Palsy) and positive serology (Table 1). Serology was assessed with the two tier testing algorithm and considered positive if (a) the first tier enzyme immunoassay was positive or equivocal and (b) the second tier IgG or IgM western blot was positive (5/10 bands and 2/3 bands for IgG and IgM, respectively). According to CDC recommendation, a positive IgM western blot is valid only for early disease, that is, in the first month of illness [33]. 24/24 patients exhibiting a characteristic cutaneous infection (EM rash) at the time of urine collection, were positive for urinary OspA peptides containing the C-terminal domain recognized by the anti-OspA mAb (Figs. 3b, 9c). Most of the positive OspA peptide bands were in the range of 28–32 kDA, but in some cases smaller fragments and a higher molecular weight band (that could be competed) in the range of 20–28 and ~60 kDa were noted (Fig. 9b). Thus the urinary shedding of OspA is likely to include OspA antigen modified by proteases. In contrast, none of 117 untreated, control patients shed urinary OspA C-terminal antigen who were non symptomatic (Chi squared p value <2.2e^−16^; significance = 5 %, power 100 %). Samples classified negative for OspA did not have detectable bands in the 20–32 kDa range (Fig. 9d). For the 24 patients that were EM positive and also urinary OspA positive at the time of urine collection, 12 became LB serology positive by CDC criteria for early diagnosis [33], 5 were serology negative [33], 3 were serology equivocal and 4 were not done (ND, Table 2). 3 untreated patients who were LB serology negative, and EM negative, but positive for joint symptoms and fatigue, were negative (0/3) for urinary OspA (Table 2). Specificity of the urinary OspA antigen test for later serology outcome was 87.5 % (21 urinary OspA positive/24 serology positive, Chi squared p = 4.072e^−15^, Table 3).

**Table 2:** Urinary OspA results compared to serology, clinical diagnosis and treatment status of N = 168 patients suspected of having early stage LB, and healthy controls

Categories	Details	Total	OspA Pos	OspA Neg
Non Lyme	Asymptomatic (EM Neg, serology ND)	117	0	117
	Symptomatic, joint pain (EM Neg, serology Neg)	3	0	3
Untreated, clinical diagnosis of LB	EM Pos (serology: 12 Pos, 5 Neg, 3 Eq, 4 ND)	24	24	0
Antibiotic treatment for a clinical diagnosis of LB	EM rash present at the time of urine collection during the treatment course (Serology: 4 Pos, 6 Neg)	10	10	0
	Arthritis Pos, EM Neg (serology: 6 Pos)	6	6	0
	Converted to EM Neg (Serology: 4 Pos, 4 Neg, 8/8 urinary OspA Pos prior to therapy)	8	0	8

*OspA pos* urinary OspA test positive, *OspA* *neg* urinary OspA test negative, *EM* Erythema Migrans, *ND* serology not done

**Table 3:** Correlation of urinary OspA to serology CDC criteria for early stage LB

	Serology positive (symptomatic and non symptomatic, pre and post treatment)	Serology negative (symptomatic and non symptomatic, pre and post treatment)	Serology equivocal Symptom Positive for clinical diagnosis of LD	Total
OspA Pos	21	17	3	41
OspA Neg	3	124	0	127
Total	24	141	3	168

Chi square calculations were obtained including negative asymptomatic healthy controls for which serology was not done

The Nanotrap system used in this study has been successfully applied in the past to verify the presence of OspA and OspB in tick vectors [30]. Fourteen ticks (females and males collected in Pennsylvania and Virginia) were analyzed. Four out of 14 ticks gave a strong positive signal in the Nanotrap particle system. The exact same band pattern of OspA identified in the extracted tick was also seen in patient’s urine in this study (Fig. 9).

### Treated patients with a clinical diagnosis of acute LB

We evaluated the urinary OspA shedding of patients who presented to a community infectious disease clinic with onset of cutaneous or systemic symptoms and were immediately treated with antibiotics based on a clinical diagnosis of LB (Tables 1, 2, Additional file 1: Table S5). Urinary OspA was scored positive or negative as described above without knowing the clinical outcome, and then compared to the clinical diagnosis based on anonymous coded patient records. Urinary OspA was compared to LB western blot serology for each patient. A subset of the treated patients donated a urine specimen at a time in the course of antibiotic therapy when the EM rash was still present. 10/10 treated patients with a concurrent EM rash at the time of urine collection were positive for urinary OspA antigen C-terminal containing peptides. Of these 10, four (4/10) post treatment were LB serology positive. Six of six (6/6) patients undergoing antibiotic therapy for a clinical diagnosis of LB who had systemic symptoms such as joint pain or neurologic symptoms, but were EM negative, were also positive for urinary OspA. All of these patients became serology positive by CDC criteria [33]. 8 patients who were initially positive for urinary OspA antigen were clinically diagnosed as symptom free (resolution of EM rash) after a successful course of antibiotic therapy for a clinical diagnosis of LB. None (0/8) asymptomatic post treated patients were positive for urinary OspA (Fig. 9e) and four (4/8) were subsequently LB serology positive. Thus urinary OspA antigen shedding appears, in this study group, to closely parallel the concurrent presence of symptoms.

### Treated patients under clinical evaluation for persistent or recurrent LB

Urinary OspA shedding was further evaluated in a cohort of 100 patients in a Lyme endemic geographic region who were under clinical surveillance for persistent or recurrent LB. All of these patients had been previously treated with antibiotics, and all patients had been followed because of prolonged chronic functional symptoms such as arthralgias, neurocognitive symptoms, and fatigue. All of these patients lacked a CDC criteria defined LB serology positive IgG western blot serum test at the time of urine collection [34]. According to the IDSA (Infectious Diseases Society of America) 2006 and 2010 guidelines [35, 36], “To date, there is no convincing biologic evidence for the existence of symptomatic chronic *B. burgdorferi* infection among patients after receipt of recommended treatment regimens for Lyme disease.” In contrast, according to ILADS (The International Lyme and Associated Diseases Society), the diagnosis of persistent Lyme disease is a real phenomenon and often requires clinical judgment to be characterized [34]. Due to the frequent nonspecific nature of complaints and insensitivities of diagnostic studies, the clinician is forced to weigh the risk profile of any individual presenting with what may be considered Lyme disease. This includes the risk of tick exposure and the presenting symptom complex [8, 34, 37–42]. In this study, urinary OspA scoring was performed blinded to the patient diagnosis or clinical findings. After the urine OspA scoring was completed, the clinical data was unblinded. For this special group of previously treated patients under surveillance for persistent or recurrent LB, 41/100 were positive for urinary OspA C-terminal peptides (Additional file 1: Table S6). This percentage of patients with positive urinary OspA is in keeping with the range of seven previous studies conducted in endemic areas where patients were being evaluated for suspected Lyme disease: 7–31 % active disease and 5–20 % previous Lyme disease in endemic areas [43–49].

## Discussion

### Value of a specific and sensitive urine test for Bb OspA

The goals of the study were to answer the following questions: Is *Bb* OspA antigen shed in the urine of patients with LB at early stage disease prior to the development of a positive serology? What is the urinary OspA concentration range? Is the OspA antigen containing the C-terminus epitope shed into urine as a full length protein or as fragments? Does the presence of OspA in the urine of a patient with an EM rash suspected of having LB correlate with the concurrent or later development of positive serology (western blot or ELISA)? If urinary OspA is present prior to antibiotic therapy, is it reduced or absent after successful therapy (resolution of symptoms)? What percentage of patients suspected of having persistent or recurrent LB contain urinary OspA C terminal domain antigen?

### OspA mAb specificity

In this study, the specificity of the mAb clone 0551 used in the Nanotrap test was verified in three ways: (1) peptide competition and immune affinity depletion, which revealed absolute specificity for a narrow C-terminus sequence of OspA that was conserved in all the *Borrelia burgdorferi* sensu latu species. (2) Viral and bacterial lysates of HSV, EBV, HCV, CMV, *Babesia*, and *Bartonella* tested at the same antigen concentration as the OspA *Borrelia* antigen were devoid of immunoreactivity with the mAb clone 0551and did not interfere with the recognition of OspA. This was done using the entire Nanotrap concentration system with test antigens spiked in human urine. (3) BLAST sequence analysis of HSV, EBV, HCV, CMV, *Babesia*, *Bartonella*, human genome database and non *Borrelia* spirochetes showed no significant similarity with the defined and verified C-terminus OspA epitope domain. It’s important to note that the specific domain recognized by the mAb clone 0551 is OspA236-239. This region is quite distant on the OspA molecule from the region that was shown in the past to have sequence similarity with human proteins (OspA154-173 = GSGKAKEVLKGYVLEGTLTA [50]).

Based on the conservation of the epitope OspA236-239, the specificity of our test should extend to all the pathogenic *Borrelia* species in the US and Europe (Fig. 3). In the future, it will be important to verify the sensitivity and specificity obtained in this study to geographically diverse populations.

### Shedding of OspA protein in the urine of patients with a diagnosis of early stage Lyme borreliosis

In order to achieve a test that can detect LB at the earliest stage, even before an immune response is mounted, we chose the well studied molecule OspA, but we focused on a novel epitope. *Borrelia* OspA plays a central role in the survival of the spirochete in the tick vector [51–53]. During the initial phases of a cutaneous infection following a tick bite, OspA is recognized by innate immune cell Toll-like receptor TLR2, and together with TLR8, initiates a cascade of proinflammatory cytokines, such as interleukin 1 and T-helper derived cytokines that are thought to mediate the initial inflammatory reaction [54]. OspA is upregulated in response to inflammatory cues by host-adapted Bb later in the course of disease [55–57]. In previous studies, specific complexed antibody to whole Bb and recombinant OspA were detected in 10 of 11 of the EM positive patients compared to 0 of 20 endemic area controls [20]. IgM was the predominant isotype recognizing OspA in these EM patients. Free IgM to OspA was found in half the EM cases. IgM to OspA was also detected in 10 of 10 European patients with EM who also had reactive T cells to recombinant OspA [20]. In longitudinal studies of serial serum samples from untreated patients (collected prior to the use of antibiotic therapy for Lyme disease treatment), elevation of IgG reactivity to OspA parallels the rising of the antibody response to Bb proteins [58, 59]. Animal models have shown that *Borrelia* antigens are shed into the urine following infection [21]. For example in early studies of *Borrelia* infection of mice in the wild, 76 % of 50 mice (white-footed mice *Peromyscus leucopus*) shed *Borrelia* antigens, including OspA, into the urine [21]. In keeping with animal model studies, OspA antigen shedding prior to antibiotic treatment occurred in the urine of 24/24 patients with a positive EM rash and clinical symptoms of LB (Table 2). In contrast, none of 117 untreated patients shed urinary OspA C-terminal antigen who were non symptomatic, or were non symptomatic and concurrently serology negative for LB.

### OspA antigen is shed into urine either as a full length protein, or as a fragment containing the C-Terminus domain

Pepsin fragmentation, followed by mass spectrometry sequencing and synthetic peptide competition, identified the anti-OspA mAb binding domain to reside in the C-terminus region of OspA (Fig. 2, Additional file 1: Table S1). In previous studies, IgG titers to OspA (and to a lesser extent, OspB) in untreated patients [20] correlate directly with severity of chronic Lyme arthritis, while IgG titers to the C-terminal third of OspA (OspA168–273) correlate with both severity and duration, suggesting the strongest causal link with epitopes contained within this fragment [59]. The C-terminal quarter of OspA (218-273) is also highly conserved among a large number of pathogenic species of *Borrelia* sequenced to date [22, 23]. The specific C-terminus peptide sequence (Additional file 1: Table S1) of the antigenic epitope showed no sequence homology with any human protein and did not have any sequence homology with non-*Borrelia* spirochetes. The size of the OspA antigen shed into the urine in the present study (Fig. 9) was close to the expected size of the full length OspA protein (approximately 31 kDa) which is small enough to be filtered through the kidney glomeruli. In some cases fragments of OspA containing the C-terminus fragment epitope were detected below the size of 32 kDa. The existence of these fragments may indicate in vivo degradation of the antigen, since we have established that Nanotrap particle capture fully stabilizes the captured analyte and prevents enzymatic degradation [18, 19, 60].

### Correlation of urinary OspA protein with positive Lyme borreliosis serology and persistence of symptoms

The clinical diagnosis of acute early stage LB is often based on the history of deer tick exposure and clinical evaluation of skin lesions for which the appearance is quite variable and the differentiation from arthropod bite reactions, gyrate erythemas and other erythematous skin conditions can be difficult [61]. In 10 % of patients LB is not considered and many patients are misdiagnosed (in one study 37 % [61]). The literature is mixed in relation to the incidence of this hallmark clinical feature of acute Lyme disease, ranging from as low as 25 % [4] to as high as 80 % [3]. The caveat from this observation is that a potentially substantial number of individuals presenting with acute Lyme disease will not manifest this dermatologic feature. But perhaps more likely with a nonspecific “flu-like” illness. In fact, Feder stated that “patients from Lyme disease endemic areas who have fever and fatigue, especially within a month following a deer tick bite, should be considered for empiric antibiotic therapy for early localized Lyme disease”, regardless of whether an EM rash is present [62]. We now present technology that may provide additional objective information to assist the clinical diagnosis and to monitor antigen shedding during the course of therapy.

A potential value of the urinary assay reported in this study is the evaluation of whether an initial course of therapy is sufficient to eradicate the infection. In 1994 Shadick et al. [40] evaluated 38 adult patients diagnosed with Lyme disease, having fulfilled establish serologic criteria at the time of the study. Initial antibiotic treatments ranged from 10 to 21 days of a standard regimen. Ten of 38 patients with Lyme disease reported relapses within 1 year of treatment (fatigue, persistent arthritis or arthralgias, headaches, or difficulty with memory and concentration). In 2014, Aucott, et al. [63] assessed the clinical response of 77 individuals presenting with an acute EM rash and completing a standard 3 week course of doxycycline. After 6 months, 39 % of this group had persistent functional impairment and/or persistence of new symptoms felt to be related to the acute infection. In 1999 Oksi et al. reported the clinical relapses of disseminated LB confirmed by culture and PCR, with various clinical presentations such as arthritis, neuropathy and uveitis [39]. Potential mechanisms contributing to this persistence of *B. burgdorferi* in human [39] and animal models [64–67] have been identified. These include: immune evasion via physical seclusion of Bb within immunologically protected tissue sites such as the CNS, joints and eyes [67–69], collagen-rich tissue [70], cells [71–74], and biofilms [75]; alterations in Osp profiles through antigenic variation [76–79], phasic variation [56], and alteration in Bb morphology (including cell-wall deficient forms, spherocytes and ‘cyst’ forms) [80–87]; immune modulation via alterations in complement [88–90], neutrophil and dendritic cell functioning [91, 92], and changes in cytokine and chemokine levels [93–95] and innate antibiotic tolerance of some *B. burgdorferi* populations [96]. Theoretically, Nanotrap technology would have the capacity to determine which, if any of these individuals had perpetuation of their symptoms due to ongoing infection.

Antibodies specific to *B. burgdorferi* proteins can take three to four weeks to develop [97], and early stage Lyme disease, prior to the appearance of a serologic titer, is extremely difficult to diagnose due to the low sensitivity of current diagnostic tests for *B. burgdorferi* antigen [98]. For this reason, treating physicians worry that Lyme serology is unreliable for early stage disease because the development of antibodies differs widely, especially in the early stages of the infection [99, 100]. In prior studies [61], only 43 % had a positive serology at the time of cutaneous EM positive LB diagnosis. Thus, the inflammatory reaction manifest in the border of the LB EM rash contains proliferating spirochetes and the inflammatory infiltrate is the result of innate immune recognition of OspA. By definition it would be expected that OspA protein antigen would be shed into the circulation and be concentrated in the urine for a significant time period prior to the development of a positive serology with 5 IgG bands as specified under CDC guidelines [99].

Prior to antibiotic treatment, in this study, 24/24 patients with an EM rash contained OspA protein in the urine, verified by peptide competition. Our analysis was blinded to outcome. Based on the sensitivity and dose response of the assay, the concentration range was between 1.7 and 30 pg/mL. 5 of these 24 early stage patients were serology negative and 3 had an equivocal serology at the time of urine collection and clinical diagnosis. Following antibiotic therapy of patients with a clinical diagnosis of LB, 10/14 patients with a positive serology were found to be positive for urinary OspA in this study. Following antibiotic therapy for a clinical diagnosis of LB, 10 patients in this study were serology negative. 4/10 of these post-treatment patients were negative for urinary OspA. Importantly, for 10 patients who exhibited persistence of the EM rash during the course of antibiotic therapy, 10/10 were positive for urinary OspA. In contrast 4/10 of these same patients who had the EM rash during antibiotic therapy ultimately became serology positive. Urinary OspA measurement of 8 patients for whom the therapeutic response was judged complete (absence of EM rash and absence of symptoms) following antibiotic therapy revealed that all 8 patients switched from being urinary OspA positive to urinary OspA negative (Table 2). 4 of these 8 patients were subsequently found to be serology positive for Bb infection. These data are in keeping with the correlation of urinary shed of OspA and the presence of concurrent objective symptoms (EM rash). When CDC criteria serology, and in accord with the Infectious Diseases Society of America (IDSA), was compared to urinary OspA regardless of pre or post treatment status of early stage LB, 87.5 % of serology positive were also urinary OspA positive and 88 % of serology negative were also urinary OspA negative (Chi squared p value = 4.072e^−15^; significance level = 5 %, power = 99.99 %, Table 3). The remaining 12 % of patients who were urinary OspA positive who were serology negative demonstrated positive symptoms qualifying for clinical diagnosis of LB and may not have yet mounted an antibody response.

In this study, 100 % of pre and post treatment samples that had active symptoms were found to be positive for detectable urinary OspA (40/40, Table 2). Urinary OspA outcome (positive) was significantly associated with presence of clinical symptoms (EM rash, Chi squared p value <2.2e^−16^; significance level = 5 %, power = 100 %).

Of these 40 patients, 22 were serology positive by CDC standards for early stage disease [33]. This is in keeping with prior studies showing positive serology in approximately 50 % of early stage cutaneous LB patients [61]. Early prompt treatment of LB is known to blunt the serology response because the infection and immune response is being interrupted at an early stage [101, 102].

### Urinary OspA in patients suspected of having “chronic” LB

Lyme disease is too often diagnosed after the infection is well established and the patient has raised an antibody titer against the bacteria *B. burgdorferi* [98]. Persistent LB, treatment resistant, recurrent, or a new LB is also extremely difficult to diagnose when the serologic titer is equivocal or if the patient has persistent symptoms (e.g. neurologic, arthritic, or dermatologic) in the face of therapy. Unfortunately patients with clinical history of LB (serology positive or serology inconclusive) can present with articular and neuromuscolar symptoms. Lack of response to treatment can theoretically be due to persistence of infection via one or more mechanims already discussed, such as sequestration in tissue or biofilm. Given the polymicrobial nature of tick borne illnesses, infection with one or more different pathogens is a consideration [103–113]. On the other hand, persistence of symptoms has been postulated to be the result of a new LB infection [103], or perhaps to improper diagnosis of LB and unrelated co-existing musculoskeletal morbidity or to persistence of infection in a sequestered tissue such as joint cartilage [9], or biofilm [75, 114, 115].

In this study, we evaluated the level of urinary OspA protein in 100 previously or currently treated patients with joint, neurologic, and other objective symptoms (Additional file 1: Table S6). This group of patients were being evaluated for the potential of recurrent or persistent infection with *Borrelia*. Our analysis was blinded to outcome. 41 of 100 (41 %) patients were positive for urinary OspA protein. This percentage of positive urinary OspA, assuming that it reflects a specific infection by Bb that is shedding OspA C-terminal fragments, is in keeping with prior studies. Patients evaluated in endemic LB regions who presented with arthritis and neurologic symptoms were estimated to have active and prior LB (7–31 % active disease and 5–20 % previous Lyme disease in endemic areas [43–48]). IDSA and ILADS differ in their recommendations for the clinical assessment and treatment of persistent LB. A highly specific antigen test for *Borrelia* proteins might provide new class of evidence to refine the guidelines for diagnosis and treatment of LB. It is widely acknowledged that patients suspected of having chronic Lyme borreliosis based neurologic or joint symptomatology may not truly have Lyme borreliosis, or may have other tick borne diseases. Therefore in patients who are suspected of having chronic Lyme disease, as evaluated in the present study, there has not been a means to assess the true positive patients. Most, if not all, of these patients have a negative Lyme serology by the 2 tier criteria. Thus our findings of 41 % positive patients in this population cannot be defined as a level of sensitivity and specificity, since none, or all, of these patients could actually have had an active *Borrelia* infection. Importantly, our data provides the first antigenic evidence that at least 41 % of these patients may have an active Bb infection. Therefore these data contribute significant new information to the debate about chronic Lyme disease.

## Conclusions

These data support the hypothesis that urinary OspA protein fragments containing the C-terminal domain occur prior to the development of a full IgG serology response, and urinary OspA strongly correlates with a clinical diagnosis and active clinical symptoms (e.g. EM rash positive) of early stage LB. Thus, this technology has the potential to provide clarity in the setting of individuals at risk of tick exposure and acutely presenting with either an atypical EM rash or without a rash at all, but consistent with the nonspecific findings of acute Lyme. Moreover, persistence of objective clinical manifestations in these patients was accompanied by continued shedding of urinary OspA even during the course of treatment. In contrast, after successful resolution of symptoms in promptly treated early stage LB, urinary OspA protein became undetectable (Table 2).

Antigenuria in the setting of chronic persistent symptoms may be due to new, acute infectious exposures. Alternatively, antigenuria detected in individuals with consistent, persistent symptoms would warrant consideration of an ongoing active infection, supportive of the concept of LB in the chronic active state.

PCR analysis of urinary *Borrelia*, or urinary *Borrelia* culture was not done, because of the very low sensitivity of these tests in human urine [28]. Consequently, a weakness of this study is that a true positive diagnosis of LB could only be based on the CDC clinical criteria (e.g. EM rash and other objective symptoms), and the development of a later positive serology in patients who underwent therapy at the time of the clinical diagnosis of LB. Despite this weakness, the strong correlation of urinary OspA with treatment response may offer a new class of information to assist the treating physician to determine whether a first round of therapy is successful in primary cutaneous early stage LB. In a population of patients being under surveillance for persistent or recurrent LB, the percentage of positive urinary OspA patients is in keeping with previous studies on patients estimated to actually have LB in endemic areas. It is impossible to know if urinary OspA, assuming that is indicative of Bb infection, is caused by a recurrent or new infection. Urinary OspA measurements may provide additional information to assist the clinical workup of patients under investigation of disseminated later stages of LB. We are attempting to validate the correlation of urinary OspA antigen with therapeutic response in an ongoing clinical study which extends the current findings.

## Additional file

10.1186/s12967-015-0701-z 81 % of the total protein present in Bb Lyme antigen Grade 2 (American Research Products) is constituted by OspA. A dilution of a recombinant purified OspA protein (Genecopoeia) was analyzed by SDS PAGE and silver stained (100 to 1,000 ng, *Lanes 3–7*). The band intensities were quantified using ImageJ software and a calibration curve was built (insert graph). A linear regression curve was fitted (y = 5.39x+12281, R^2^ = 0.9873). 500 ng of Bb Lyme antigen Grade 2 were loaded on the gel (*Lane 2*) and compared to the recombinant OspA calibration curve (*Lanes 3–7*). From this procedure it derived that 81% of Bb Lyme antigen Grade 2 total protein content is OspA. In gel protein digestion and mass spectrometry (MS) analysis verified the predominant presence of tryptic peptides belonging to OspA in the bands at ~30 kDa and ~60 kDa present both in the Bb Lyme antigen Grade 2 and in the recombinant OspA. In particular, tryptic peptides containing the epitope for mAb clone 0551 OspA236-239 were present in both bands (MS-MS spectra are reported in the insert). **Figure S2**. Narrow OspA236-239 region binds to mAb clone 0551 with high specificity. Dot blot analysis revealed that only 2 out of 7 synthetic peptides with sequence reported in Table S1 show reactivity with the anti OspA monoclonal antibody clone 0551 used in this study. **Figure S3**.  Light scattering analysis was used to determine the hydrodynamic diameter of the Nanotrap particles. As reported in Table S2, the hydrodynamic diameter of the Nanotrap particles was 1054.7 +/- 31.11 nm. **Figure S4**.  A V/v ratio (V = Nanotrap suspension volume, v = sample volume) of 1/10 was optimized in order to maximize Lyme antigen capturing. **a** 2 ng of Lyme antigen was spiked in 500 µL urine aliquots. Urine samples were incubated with increasing amount of Nanotrap particle suspension (10–160 µL of Nanotrap at 5 mg/mL concentration). *Lane 1* ladder; *lane 2* Positive control (OspA 1ng); *lane 3* 10 µL of Nanotrap particles; *lane 4* 20  µL Nanotrap particles; *lane 5* 40 µL Nanotrap particles; *lane 6* 80 µL Nanotrap particles; *lane 7* 160 µL Nanotrap particles; **b **Band intensity was measured with ImageJ, plateau is reached with >40 µL of Nanotrap particles. **Figure S5**. Interfering substances: the presence of a high amount of protein and blood in the urine does not interfere with Lyme antigen capture and detection. **a** 320 pg of Lyme antigen was spiked in samples of 40 mL of human urine. We tested the interference of albumin present in excess up to 10^8 fold. Increasing amounts of bovine serum albumin ranging from 0.31 mg/mL to 20 mg/mL were added to 40 mL of OspA containing human urine; urine samples were processed with Nanotrap particles. Ability of the Nanotrap particles to sequester OspA is not affected by increasing concentration of competing proteins in urine.* Lane 1* ladder; *lane 2* volunteer human urine in absence of OspA antigen (negative control); *lane 3* OspA + BSA 20 mg/mL; *lane 4* OspA + BSA 10 mg/mL; *lane 5* OspA + BSA 5 mg/mL; *lane 6* OspA + BSA 2.5 mg/mL; *lane 7* OspA + BSA 1.25 mg/mL; *lane 8* OspA + BSA 0.625 mg/mL; *lane 9* OspA + BSA 0.31 mg/mL;* lane 10 *OspA + BSA 0.15 mg/mL. **b** Lyme antigen 320 pg was spiked in urine samples (40 mL). Increasing amounts of whole blood from 0.015 µL to 1 µL was added to the urine samples; urine samples were processed with Nanotrap particles and analyzed using western blot. *Lane 1* ladder; *lane 2* volunteer human urine in absence of OspA antigen (negative control); *lane 3* OspA + 1 µL whole blood; *lane 4* OspA + 0.5 µL whole blood; *lane 5* OspA + 0.25 µL whole blood; *lane 6* OspA + 0.125 µL whole blood; *lane 7* OspA + 0.062 µL whole blood; *lane 8* OspA + 0.031 µL whole blood; *lane 9* OspA + 0.015 µL whole blood; *lane 10 *OspA + 0.007 µL whole blood. **Figure S6. a** Nanotrap particle preprocessing step is necessary to detect an OspA specific band in the urine of patient PD113, clinically positive for Lyme disease. Initial solution (IS) before Nanotrap particle processing. Eluate (E) after Nanotrap particle processing. **b **Positive and negative controls run with all Lyme patient samples. *Lane 2* contains borrelia protein lysate (2 ng) in human urine. *Lane 5 *is a positive control 4 ng spiked in 40 mL of urine sample. *Lane 6 *and *7* are negative controls of 40 mL of volunteer urine processed through the Nanotrap particles. C=borrelia lysate control, IS=initial solution, S=supernatant, E=eluate. **Table S1**. Sequences of peptides tested for antibody binding. **Table S2**. The mAb epitope used herein (red rectangle) is conserved in common pathogenic species of Borrelia. BAA22342.1 in Borrelia garinii Taxonomy ID 29519, ADD14639.1 in Borrelia burgdorferi taxonomy ID 139, WP_012665647.1 in Borrelia valaisiana taxonomy ID 62088, WP_012665647.1 in Borrelia sp. SV1 taxonomy ID 498741, YP_003110622.1 in Borrelia burgdorferi 297 taxonomy ID 521009, NP_045688.1 in Borrelia burgdorferi B31 taxonomy ID 224326, WP_014023199.1 in Borrelia bissettii taxonomy ID 64897, ADG02035.1 in Borrelia afzelii taxonomy ID 29518, AAN65460.1 in Borrelia spielmanii taxonomy ID 88916. BLAST analysis was performed on the sequence KTSTLTISVNSKTTQLVFTKQDTITVQKYDSAGT (combination of peptide 5 and 6) with the following organisms: Treponema pertenue (taxonomy ID 168), Leptospiraceae (taxonomy ID 170), Treponema (taxonomy ID 157), Spirochaetes (taxonomy ID 203691) excluding Borrelia (taxonomy ID 138), Homo sapiens (taxonomy ID 9606), Epstein-Barr virus EBV (taxid:10376), Human cytomegalovirus (taxid:10359), herpes simplex virus 1 HSV-1 (taxid:10298), hepatitis C virus HCV (taxid:11103), Babesia txid5864, Anaplasma txid768, Ehrlichieae txid942, Bartonella  txid773, Rickettsias txid766.  No significant similarity found.  No homology was identified. **Table S3.** Light scattering analysis of Nanotrap particles. **Table S4**. Quantification of the amount of Remazol brilliant blue (RBB) dye covalently bound to the Nanotrap particles and percentage of reacted acrylic acid (AAc) moles. This information was obtained and recorded for every batch of produced Nanotrap particles (example batch # RM37B4 is reported here). **Table S5.** Clinical and diagnostic information of patients suspected of having early stage Lyme disease N=51 (N= 117 healthy volunteers were recruited under informed consent and included in the study). Treatment: Dx = doxycycline, Pd = prednisone, RC = rocepherin, sv = synovectomy, st = steroids, Am = amoxil, Zm = Zithromax. Urine collection timing and presence of symptoms: B = before treatment, PT = during or after treatment, symptoms present at the time urine was collected. **Table S6.** Post treatment patients being evaluated for recurrent or persistent disseminated Lyme disease derived from a Lyme endemic geographic region.

## Abbreviations

AAc: crylic acid
AU: arbitrary units
Bb: *Borrelia burgdorferi*
BIS: *N*, *N*′-methylenebisacrylamide
CDC: centers for disease control
CV: coefficient of variation
EIA: enzyme immunoassay
EM: erythema migrans
IRB: internal review board
LB: Lyme borreliosis
LLB: lower limit of detection
LLQ: lower limit of quantification
mAb: monoclonal antibody
NIPAm: *N*-isopropylacrylamide
OspA: outer surface protein A
PCR: polymerase chain reaction
PDB: protein data bank
SD: standard deviation
TLR2: toll like receptor 2
TLR8: toll like receptor 8
