# Supplementary material for: Application of Nanotrap technology for high sensitivity measurement of urinary outer surface protein A carboxyl-terminus domain in early stage Lyme borreliosis
Source: J Transl Med. 2015 Nov 4;13:346. doi: 10.1186/s12967-015-0701-z (PMC4634744; doi:10.1186/s12967-015-0701-z)
Supplement: Supplementary file 1 — 10.1186/s12967-015-0701-z 81 % of the total protein present in Bb Lyme antigen Grade 2 (American Research Products) is constituted by OspA. A dilution of a recombinant purified OspA protein (Genecopoeia) was analyzed by SDS PAGE and silver stained (100 to 1,000 ng, Lanes 3–7). The band intensities were quantified using ImageJ software and a calibration curve was built (insert graph). A linear regression curve was fitted (y = 5.39x+12281, R2 = 0.9873). 500 ng of Bb Lyme antigen Grade 2 were loaded on the gel (Lane 2) and compared to the recombinant OspA calibration curve (Lanes 3–7). From this procedure it derived that 81% of Bb Lyme antigen Grade 2 total protein content is OspA. In gel protein digestion and mass spectrometry (MS) analysis verified the predominant presence of tryptic peptides belonging to OspA in the bands at ~30 kDa and ~60 kDa present both in the Bb Lyme antigen Grade 2 and in the recombinant OspA. In particular, tryptic peptides containing the epitope for mAb clone 0551 OspA236-239 were present in both bands (MS-MS spectra are reported in the insert). Figure S2. Narrow OspA236-239 region binds to mAb clone 0551 with high specificity. Dot blot analysis revealed that only 2 out of 7 synthetic peptides with sequence reported in Table S1 show reactivity with the anti OspA monoclonal antibody clone 0551 used in this study. Figure S3. Light scattering analysis was used to determine the hydrodynamic diameter of the Nanotrap particles. As reported in Table S2, the hydrodynamic diameter of the Nanotrap particles was 1054.7 +/- 31.11 nm. Figure S4. A V/v ratio (V = Nanotrap suspension volume, v = sample volume) of 1/10 was optimized in order to maximize Lyme antigen capturing. a 2 ng of Lyme antigen was spiked in 500 µL urine aliquots. Urine samples were incubated with increasing amount of Nanotrap particle suspension (10–160 µL of Nanotrap at 5 mg/mL concentration). Lane 1 ladder; lane 2 Positive control (OspA 1ng); lane 3 10 µL of Nanotrap particles; lane [file 12967_2015_701_MOESM1_ESM.pdf]

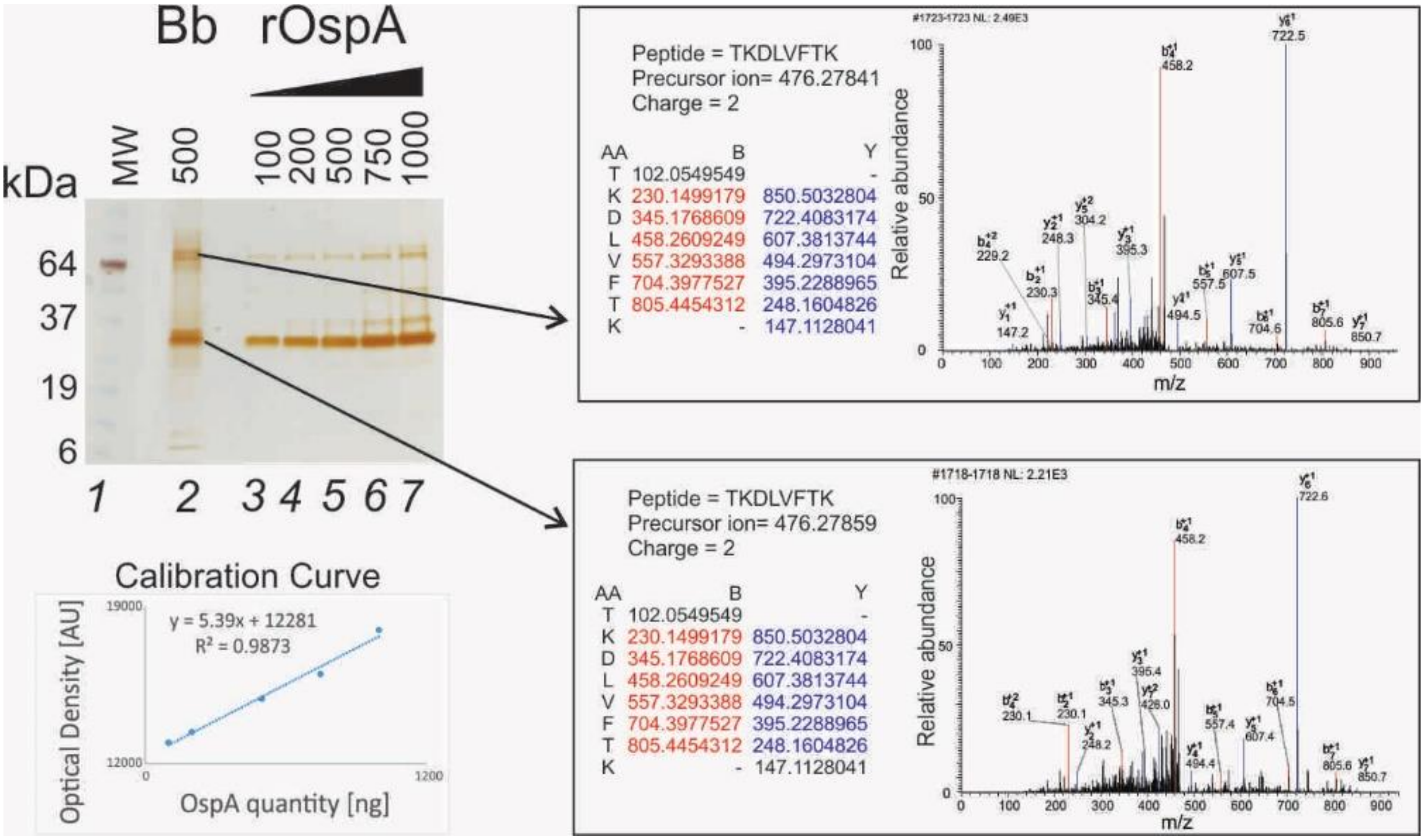

Figure S1: 81% of the total protein present in Bb Lyme antigen Grade 2 (American Research Products) is constituted by OspA. A dilution of a recombinant purified OspA protein (Genecopoeia) was analyzed by SDS PAGE and silver staining (100-to 1,000 ng, *Lanes 3-7*). The band intensities were quantified using ImageJ software and a calibration curve was built (insert graph). A linear regression curve was fitted (y=5.39x+12281, R^2 = 0.9873). 500 ng of Bb Lyme antigen Grade 2 were loaded on the gel (*Lane 2*) and compared to the recombinant OspA calibration curve (*Lanes 3-7*). From this procedure it derived that 81% of Bb Lyme antigen Grade 2 total protein content is OspA. In gel protein digestion and mass spectrometry (MS) analysis verified the predominant presence of tryptic peptides belonging to OspA in the bands at ~30 kDa and ~60kDa present both in the Bb Lyme antigen Grade 2 and in the recombinant OspA. In particular, tryptic peptides containing the epitope for mAb clone 0551 OspA236-239 were present in both bands (MS-MS spectra are reported in the insert).

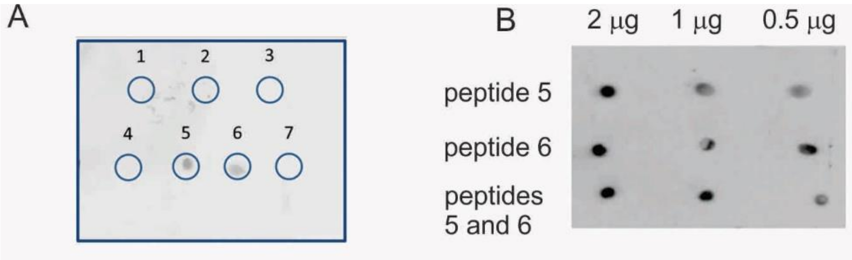

C

Peptide Sequences

OspA peptide 1	KQNVSSLDEKNSASVDLPGE
OspA peptide 2	VLKNFTLEGKVANDKTLEV
OspA peptide 3	EVKEGTVTLSKEIAKSGEVT
OspA peptide 4	VALNDTNTTQATKKTGAWDS
OspA peptide 5	KTSTLTISVNSKKTTQL**VFTK**
OspA peptide 6	QL**VFTK**QDTITVQKYDSAGT
OspA peptide 7	NLEGTAVEIKTLDELKNALK

Figure S2. Narrow OspA236-239 region binds to mAb clone 0551 with high specificity. Dot blot analysis revealed that only 2 out of 7 synthetic peptides with sequence reported in Table S1 show reactivity with the anti OspA monoclonal antibody clone 0551 used in this study.

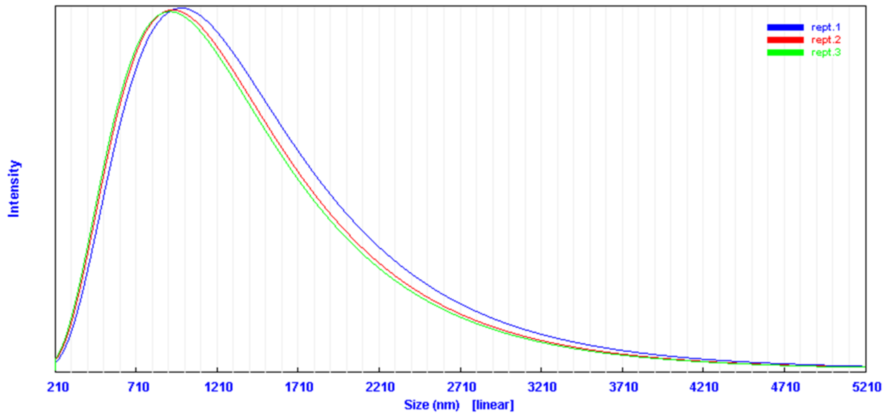

Figure S3. Light scattering analysis was used to determine the hydrodynamic diameter of the Nanotrap particles. As reported in Table S2, the hydrodynamic diameter of the Nanotrap particles was 1054.7 +/- 31.11 nm.

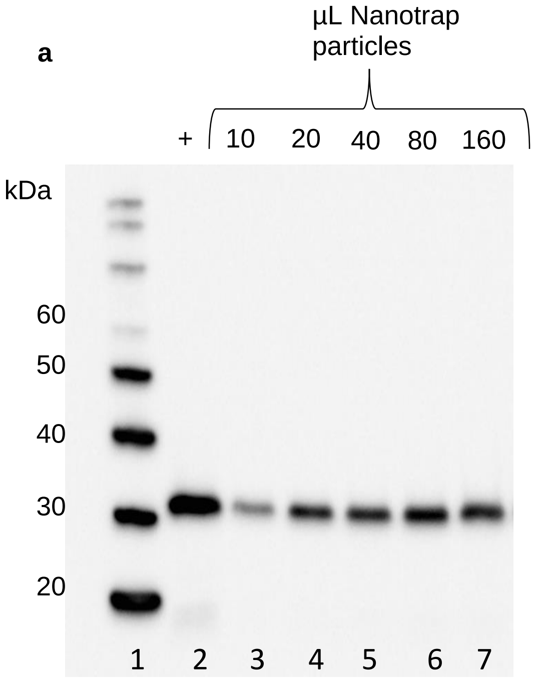

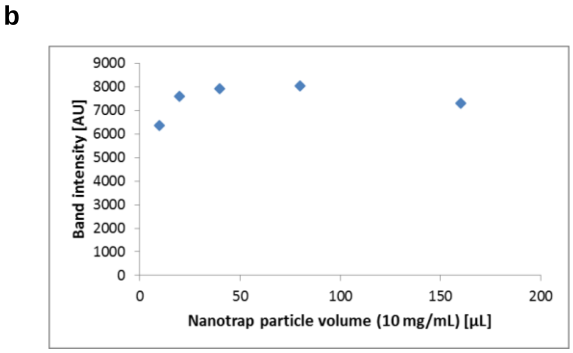

Figure S4. A V/v ratio (V=Nanotrap suspension volume, v=sample volume) of 1/10 was optimized in order to maximize Lyme antigen capturing. **a** 2 ng of Lyme antigen was spiked in 500 μL urine aliquots. Urine samples were incubated with increasing amount of Nanotrap particle suspension (10-160 μL of Nanotrap at 5 mg/mL concentration). *Lanes 1*) ladder; *2*) Positive control (OspA 1ng); *3*) 10μL of Nanotrap particles; *4*) 20 μL Nanotrap particles; *5*) 40μL Nanotrap particles; *6*) 80 μL Nanotrap particles; *7*) 160μL Nanotrap particles; **b** Band intensity was measured with ImageJ, plateau is reached with > 40μL of Nanotrap particles.

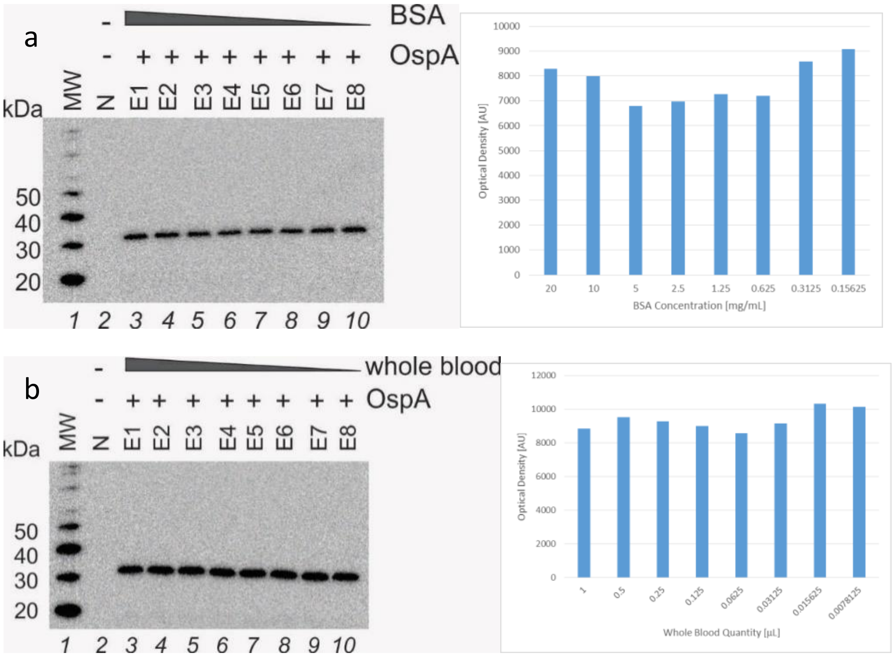

Figure S5. Interfering substances: the presence of a high amount of protein and blood in the urine does not interfere with Lyme antigen capture and detection. **a** 320 pg of Lyme antigen was spiked in samples of 40 mL of human urine. We tested the interference of albumin present in excess up to 10^8 fold. Increasing amounts of bovine serum albumin ranging from 0.31 mg/mL to 20 mg/mL were added to 40 mL of OspA containing human urine; urine samples were processed with Nanotrap particles. Ability of the Nanotrap particles to sequester OspA is not affected by increasing concentration of competing proteins in urine. *Lanes*: *1*) ladder; *2*) volunteer human urine in absence of OspA antigen (negative control); *3*) OspA + BSA 20 mg/mL ; *4*) OspA + BSA 10 mg/mL; *5*) OspA + BSA 5 mg/mL; *6*) OspA + BSA 2.5 mg/mL; *7*) OspA + BSA 1.25 mg/mL; *8*) OspA + BSA 0.625 mg/mL; *9*) OspA + BSA 0.31 mg/mL; *10*) OspA + BSA 0.15 mg/mL. **b** Lyme antigen 320 pg was spiked in urine samples (40 mL). Increasing amounts of whole blood from 0.015 µL to 1 µL was added to the urine samples; urine samples were processed with Nanotrap particles and analyzed using western blot. Lanes: *1*) ladder; *2*) volunteer human urine in absence of OspA antigen (negative control); *3*) OspA + 1 µL whole blood; *4*) OspA + 0.5 µL whole blood; *5*) OspA + 0.25 µL whole blood; *6*) OspA + 0.125 µL whole blood; *7*) OspA + 0.062 µL whole blood; *8*) OspA + 0.031 µL whole blood; *9*) OspA + 0.015 µL whole blood; *10*) OspA + 0.007 µL whole blood.

Note: Bilirubin, which we screen against in the urine against in the urine dipstick prior to analysis, does not cause any interference with the Nanotrap based test for the following two reasons. Firstly, the Nanotrap particle urine OspA test uses chemiluminescence solid phase based detection whereas bilirubin is known to interfere with absorbance readings at λ ~ 456 nm [Clin Biochem Rev Vol 29 Suppl (i) August 2008 I S43] in solution-phase homogeneous assays. Secondly, the molecular weight of bilirubin is ~500 Da. This means that bilirubin is too small to be retained in the SDS PAGE system (Tris Gly 4-20%) we use to detect the OspA antigen. Bilirubin would migrate out of the gel during the electrophoretic run necessary to separate the proteins, which have a much higher molecular weight (10,000 – 100,000 Da). The presence of abnormally high proteins in the urine of patients, as happens in the in the rare disorder of monoclonal gammopathy (3% incidence in general population >50 yo, decreasing with decreasing age), does not interfere with the Nanotrap OspA test as demonstrated in

Figure S5. The exquisite specificity of the test is ensured by the specificity of the anti OspA monoclonal antibody and by the two-step test that includes a competition assay for each positive result. This competition assures that immunoreactivity with the antigen can be clearly differentiated from the rare possibility of background caused by immunoglobulins in the urine.

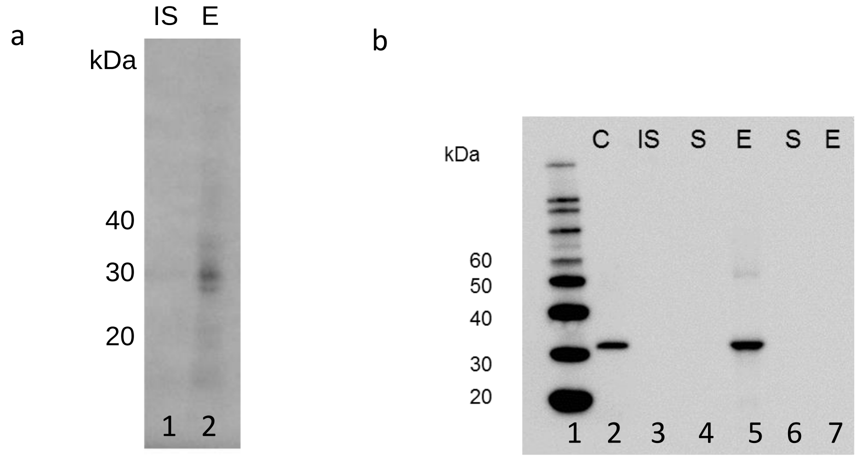

Figure S6. a Nanotrap particle preprocessing step is necessary to detect an OspA specific band in the urine of patient PD113, clinically positive for Lyme disease. Initial solution (IS) before Nanotrap particle processing. Eluate (E) after Nanotrap particle processing. **b** Positive and negative controls run with all Lyme patient samples. *Lane 2* contains borrelia protein lysate (2 ng) in human urine. *Lane 5* is a positive control 4 ng spiked in 40 mL of urine sample. *Lane 6* and *7* are negative controls of 40 mL of volunteer urine processed through the Nanotrap particles. C=borrelia lysate control, IS=initial solution, S=supernatant, E=eluate.

Table S1. Sequences of peptides tested for antibody binding.

Peptide ID	Sequence	Reactivity with mAb 0551
OspA peptide 1	KQNVSSLDEKNSASVDLPGE	Negative
OspA peptide 2	VLKNFTLEGKVANDKTLEV	Negative
OspA peptide 3	EVKEGTVTLSKEIAKSGEVT	Negative
OspA peptide 4	VALNDTNTTQATKKTGAWDS	Negative
OspA peptide 5	KTSTLTISVNSKKTTQL**VFTK**	Positive
OspA peptide 6	QL**VFTK**QDTITVQKYDSAGT	Positive
OspA peptide 7	NLEGTAVEIKTLDELKNALK	Negative
OspA219-253	KTSTLTISVNSKKTTQL**VFTK**QDTITVQKYDSAGT	Positive
OspA219-235	KTSTLTISVNSKKTTQL	Negative
OspA240-253	QDTITVQKYDSAGT	Negative

Table S2. The mAb epitope used herein (red rectangle) is conserved in common pathogenic species of *Borrelia*. BAA22342.1 in *Borrelia garinii* Taxonomy ID 29519, ADD14639.1 in *Borrelia burgdorferi* taxonomy ID 139, WP_012665647.1 in *Borrelia valaisiana* taxonomy ID 62088, WP_012665647.1 in *Borrelia* sp. SV1 taxonomy ID 498741 YP_003110622.1 in *Borrelia burgdorferi* 297 taxonomy ID 521009, NP_045688.1 in *Borrelia burgdorferi* B31 taxonomy ID 224326, WP_014023199.1 in *Borrelia bissettii* taxonomy ID 64897, ADG02035.1 in *Borrelia afzelii* taxonomy ID 29518, AAN65460.1 in *Borrelia spielmanii* taxonomy ID 88916. BLAST analysis was performed on the sequence KTSTLTISVNSKTTQLVFTKQDTITVQKYDSAGT (combination of peptide 5 and 6) with the following organisms: *Treponema pertenue* (taxonomy ID 168), *Leptospiraceae* (taxonomy ID 170), *Treponema* (taxonomy ID 157), *Spirochaetes* (taxonomy ID 203691) excluding *Borrelia* (taxonomy ID 138), *Homo sapiens* (taxonomy ID 9606), Epstein-Barr virus EBV (taxid:10376), Human cytomegalovirus (taxid:10359), herpes simplex virus 1 HSV-1 (taxid:10298), hepatitis C virus HCV (taxid:11103), Babesia txid5864, Anaplasma txid768, Ehrlichieae txid942,Bartonella txid773, Rickettsias txid766. No significant similarity found. No homology was identified.

```
BAA22342.1       1     -----LGIGLILALIACKQNVSSLDEKNSVSVDLPGEMKVLVSKEKDKDGKYSLMA
ADD14639.1       1     ----------ILALIACKQNVSSLDEKNSVSVDLPGEMKVLVSKEKNKDGKYDLIA
WP_012672372.1   1     MKKYLLGIGLILALIACKQNVSSLDEKNSVSVDLPGEIKVLVSKEKNKDGKYSLMA
WP_012665647.1   1     MKKYLLGIGLILALIACKQNVSSLDEKNSASVDLPGEMKVLVSKEKDKDGKYSLVA
YP_003110622.1   1     MKKYLLGIGLILALIACKQNVSSLDEKNSVSVDLPGEMKVLVSKEKNKDGKYDLIA
NP_045688.1      1     MKKYLLGIGLILALIACKQNVSSLDEKNSVSVDLPGEMKVLVSKEKNKDGKYDLIA
WP_014023199.1   1     MKKYLLGIGLILALIACKQNVSGLDEKNSVSVDLPGEMKVLVSKEKDKDGKYSLMA
ADG02035.1       1     ------------ALIACKQNVSSLDEKNSASVDLPGEMKVLVSKEKDKDGKYSLKA
AAN65460.1       1     ----------ILALIACKQNVSGLDEKNSTSVDVPGELKVLVSKEKDKDGKYSLMA

BAA22342.1       52    TVDKLELKGTSDKSNGSGILEGVKTDKSKAKLTISDDLSKTTFEVFKEDGKTLVSR
ADD14639.1       47    TVDKLELKGTSDKNNGSGVLEGVKADKSKVKLTISDDLGQTTLEVFKEDGKTLVSK
WP_012672372.1   57    TVDKLELKGTSDKNNGSGVLEGVKADKSKVKLTVSDDLGQTTLEVLKEDGKTLVSR
WP_012665647.1   57    TVDKVELKGTSDKNNGSGTLEGVKDDKSKVKLTISDDLGETKLETFKEDG-TLVSR
YP_003110622.1   57    TVDKLELKGTSDKNNGSGVLEGVKADKSKVKLTISDDLGQTTLEVFKEDGKTLVSK
NP_045688.1      57    TVDKLELKGTSDKNNGSGVLEGVKADKSKVKLTISDDLGQTTLEVFKEDGKTLVSK
WP_014023199.1   57    TVDKLELKGTSDKNNGSGILEGVKADKSKVKLTVSEDLSTTTLEVLKEDGKTLVSK
ADG02035.1       45    TVDKIELKGTSDKDNGSGVLEGTKDDKSKAKLTIADDLSKTTFELFKEDGKTLVSR
AAN65460.1       47    TVDKLELKGTSDKNDGSGVLEGVKADKSKVKLTI SDHLSKTTFEVFKEDGKTLVSR

BAA22342.1       108   KVNSKDKSSTEEKFNAKGELSEKVVTRANGNRLEYTEI-KSDGSGKAKEVLKDFTL
ADD14639.1       103   KVTSKDKSSTEEKFNEKGEVSEKIITRADGTRLEYTEI-KSDGSGKAKEVLKG-YV
WP_012672372.1   113   KVTSKDKSSTEEKFNEKGELAEKIMTRANGTRLEYTEI-KSDGSGKAKEVLKD-YV
WP_012665647.1   112   KVNFKDKSFTEEKFNEKGEVSEKILTRSNGTTLEYSQMtDAENATKAVETLKNgIK
YP_003110622.1   113   KVTSKDKSSTEEKFNEKGEVSEKIITRADGTRLEYTEI-KSDGSGKAKEVLKG-YV
NP_045688.1      113   KVTSKDKSSTEEKFNEKGEVSEKIITRADGTRLEYTGI-KSDGSGKAKEVLKG-YV
WP_014023199.1   113   KTTSKDKSSTEEKFNDKGELAEKTIVRANGTRLEYTEV-KSDGSGKAKETLKD-YA
ADG02035.1       101   KVSSKDKTSTDEMFNEKGELSAKTMTRENGTKLEYTEM-KSDGTGKAKEVLKN-FT
AAN65460.1       103   NVNSKDKSSTKEKFNEKGELSEKTLVRANGTKLEYTEI-KSDGTGKAKEVLKD-FT

BAA22342.1       163   LEGTLTADKTTLTIQEGSVTLSKNIAKSGEITVELNDTDSS-GDKKSGQWNSSTST
ADD14639.1       157   LEGTLTAEKTTLVVKEGTVTLSKNISKSGEVSVELNDTDSSAATKKTAAWNSGTST
WP_012672372.1   167   LEGTLTAEKTTLVVKEGTVTLSKHISKSGEVTAELNDTESSSATKKTAAWNSGTST
WP_012665647.1   168   LPGNLVGGKTTLKITEGTVTLSKHIAKSGEVTVEINDTSSTPNTKKTGKWDARNST
YP_003110622.1   167   LEGTLTAEKTTLVVKEGTVTLSKNISKSGEVSVELNDTDSSAATKKTAAWNSGTST
NP_045688.1      167   LEGTLTAEKTTLVVKEGTVTLSKNISKSGEVSVELNDTDSSAATKKTAAWNSGTST
WP_014023199.1   167   LEGTLTAEKATLVVKEGTVTLSKHISKSGEVTAELNDTDSAQATKKTGKWDAGTST
ADG02035.1       155   LEGKVANDKVTLEVKEGTVTLSKEIAKSGEVTVALNDTNTTQATKKTGAWDSKTST
AAN65460.1       157   LEGTLANEKATLTVKEGTVTLSKNIDKSGEVTVALNDTDSTAATKKTGAWDSKTST

BAA22342.1       218   LTISAKSKKTKDLVFTKQDTITVQKYDSAGTNLEGSAVEIKTLDELKNALK
ADD14639.1       213   LTITVNSKKTKDLVFTKENTITVQQYDSNGTKLEGSAVEITKLDEIKNALR
WP_012672372.1   223   LTITVNSKKTKDLVFTKENTITVQKYDTAGTNLEGSAVEIKKLDELKNALK
WP_012665647.1   224   LTIIVDSKNKTKLVFTKQDTITVQSYNPAGNKLEGTAVEIKTLQELKNALK
YP_003110622.1   223   LTITVNSKKTKDLVFTKENTITVQQYDSNGTKLEGSAVEITKLDEIKNALK
NP_045688.1      223   LTITVNSKKTKDLVFTKENTITVQQYDSNGTKLEGSAVEITKLDEIKNALK
WP_014023199.1   223   LTISVNSKKTKNLVFTKQDTITVQKYDSAGTNLEGTAVEIKTLDELKNALK
ADG02035.1       211   LTISVNSKKTTQLVFTKQDTITVQKYDSAGTNLEGTAVEIKTLDELKNA--
AAN65460.1       213   LTITVNSKKTKDLVFTKQDTITVQKYDSAGTNLEGSAVEIK----------
```

Table S3. Light scattering analysis of Nanotrap particles

Rept#	Mean [nm]	P.I.	Diff.Coef [m^2/s]	Counts/s	Baseline error
1	1097.4	-1.065	4.47e-13	1.02e+5	4.50%
2	1042.8	-1.230	4.70e-13	1.01e+5	1.21%
3	1024.0	-1.422	4.79e-13	9.95+4	0.33%
Average	1054.7 +/- 31.11	-1.239 +/- 0.146			

Table S4. Quantification of the amount of Remazol brilliant blue (RBB) dye covalently bound to the Nanotrap particles and percentage of reacted acrylic acid (AAc) moles. This information was obtained and recorded for every batch of produced Nanotrap particles (example batch # RM37B4 is reported here).

**Parameter**	**Numerical value**	**Unit of Measure**
Weight of 20 mL of freezed dried poly(NIPAm-co-AAc) Nanotrap*	110	mg
Weight of 20 mL of freeze dried poly(NIPAm/RBB) Nanotrap	153	mg
Δ weight	43	mg
Molar quantity of RBB covalently bound to the Nanotrap	0.0686	mmol
Molar quantity of AAc in the poly(NIPAm-co-AAc) Nanotrap	0.2430	mmol
Percentage of mol RBB bound to AAc / tot mol AAc	28	%

*Note: The weight is relative to the poly(NIPAm-co-AAc) Nanotrap before RBB covalently binding.

Table S5. Clinical and diagnostic information of patients suspected of having early stage Lyme disease N=51 (N= 117 healthy volunteers were recruited under informed consent and included in the study).
Treatment: Dx = doxycycline, Pd = prednisone, RC = rocepherin, sv = synovectomy, st = steroids, Am = amoxil, Zm = Zithromax. Urine collection timing and presence of symptoms: B = before treatment, PT = during or after treatment, symptoms present at the time urine was collected.

Patient ID#	Clinical manifestation	Treatment	Serology results (ELISA, IgG, IgM) N=negative, P=positive, ND=not done	Urine collection timing	OspA urine test (N=negative; P=positive confirmed by competition)
1	joint pain, malaise, neck pain, headache, nausea, dizziness	Dx	N, ND, ND	PT	N
3	EM, bell's palsy, myalgia	Dx, Pd	P, N, N	PT	P
8	myalgia, fever, joint pain	Dx, Rc, Sv	P, P, N	PT	P
11	EM, Bell's palsy, joint pain	Dx, Rc	N, ND, ND	PT	P
13	EM, fever, joint pain, neck pain, fatigue	Dx	P, N, P	PT	P
14	joint pain	St, Dx	P, P, ND	PT	P
15	EM, myalgia, fever, joint pain, malaise, fatigue	Rc, Pc	P, N, P	PT	P
17	joint pain	Dx, Rc	P, P, N	PT	P
18	EM, fever, joint pain	Dx	P, P, P	PT	P
21	headaches	Dx	N, N, N	PT	P
23	EM, fever, anthralgiatis	Am	P, P, P	B	P
24	EM, fever, anthralgias	Zm	P, N, P	PT	P
25	EM, fever, neck pain, fatigue	Dx	P, N, P	B	P
26	EM, anthralgiatis	Dx, Rc, Am	N, N, N	PT	P
27	EM, fatigues, anthralgiatis	Dx, Zm	ND, ND, ND	PT	P
102	EM		N, ND, ND	PT	P
103	fatigue, joint pain, pos for Lyme on lumber puncture	Dx	P, P, N	PT	P
103.1	No symptoms	Rc	P, P, N	-	N
105	joint pain, arthritis	Dx	P, N, N	PT	P

108	EM, fever, joint pain	Dx	P, N, P	B	P
108.1	no symptoms	Dx	P, N, P	-	N
113	EM, fever, neck pain, fatigue		P, N, P	PT	P
116	joint paint, fatigue		N, ND, ND	-	N
117	Tick bite		N, NA	B	P
118	EM fatigue		P, P, P	B	P
119	joint pain, arthritis, fatigue, neurologic	Dx	P, N, P	PT	P
120	EM, fever, malaise	Dx	P, N, P	B	P
120.1	No symptoms	Dx	P, N, P	-	N
121	EM	Dx	N, NA, NA	B	P
133	joint pain, fever, fatigue		N, N, N	-	N
139	joint pain, fatigue	Dx	P, N, P	B	P
139.1	No symptoms		P, N, P	-	P
142	rash joint pain		P, N, P	B	P
148	joint pain, fatigue		P, P, P	B	P
151	fatigue	Dx	P, N, N	PT	P
169	EM, neck stiffness, migraine		Equivocal	B	P
180	EM, tick bite	Dx	N, N, N	B	P
180.1	no symptoms	Dx	P, N, P	-	N
601	EM	Az	Equiv	B	P
601.1	no symptoms	Az	Equiv	-	N
602	EM	Dx	N, ND, ND	B	P
603	EM	Dx	P, P, P	B	P
604	EM	Dx	ND	B	P
605	EM	Dx	ND	B	P
606	EM	Dx	ND	B	P
607	EM	Dx	Equiv	B	P
608	EM	Dx	ND	B	P
623	EM	Dx	N, ND, ND	B	P
623.1	EM	Dx	N, ND, ND	-	P
623.2	EM	Dx	N, ND, ND	-	N
623.3	EM	Dx	N, ND, ND	-	N
623.4	EM	Dx	N, ND, ND	-	N
623.5	EM	Dx	N, ND, ND	-	N

Table S6: Post treatment patients being evaluated for recurrent or persistent disseminated Lyme disease derived from a Lyme endemic geographic region.

Patient ID#	Clinical Manifestation	Serology results (IgG, IgM)	OspA urine test (N=negative; P=positive confirmed by competition)
300	Neurocognitive	ND	N
306	Neurologic	ND	N
309	Neurologic	ND	N
310	Joint pain	ND	N
315	Joint pain	ND	P
318	Joint pain	ND	N
319	Bladder pain	ND	N
320	Neurologic	ND	N
323		ND	P
324	asymptomatic	ND	N
327	Bladder pain	ND	N
328	Bladder pain	ND	P
329	Joint pain	ND	N
331	neurologic	ND	N
333	Bladder pain	ND	N
334	Joint pain	ND	P
336	Fatigue	ND	N
337	Joint pain	ND	P
339	neurologic	ND	P
340	Neurologic	ND	P
341	Joint pain	ND	P
343	Myalgia	ND	N
344	Neurolocognitive	ND	N
346	EM	N,P	P
347	Neurologic	ND	P
348	Neurologic	ND	N
349	Neurocognitive	ND	P
350	Neurologic	ND	P
353	Neurologic	ND	N
355	Neurologic	ND	N
356	Neurologic	ND	P
360	Joint pain	ND	P
361	fatigue	ND	N
363	Joint pain	ND	N
364	Joint pain	ND	N
377	Joint pain	ND	N
378	Neurologic	ND	N
379	Neurologic	ND	P
380	Fatigue	ND	P
381	Joint pain	ND	P

382	Joint pain	ND	P
383	Neurologic	ND	P
384	Joint pain	N,N	P
386	Joint pain	ND	N
388	Joint pain, fatigue	ND	P
389	Joint pain	ND	P
390	Joint pain	N,N	P
391	Joint pain	N, P	N
392	Joint pain	N, N	N
393	Joint pain	ND	P
394	Joint pain	N, N	N
397		ND	N
400	EM	N, N	N
401		ND	N
402		ND	N
403		ND	N
404		ND	N
406	Neurocognitive	ND	N
407	Joint pain	N,N	P
410		N,N	P
413		ND	N
414	Joint pain	ND	N
416		ND	N
417		ND	N
419		ND	P
421		ND	N
424	Neurologic	N,N	N
425		N,N	P
426		ND	N
427	EM, joint pain	N,N	P
429	Neurologic	ND	N
430	Neurocognitive	ND	N
431	Neurologic	ND	N
433	Joint pain	ND	N
435	Neurocognitive	ND	N
437		ND	P
438	Joint pain	ND	P
439	Joint pain	ND	P
443	Myocarditis	ND	P
446	Neurocognitive	ND	N
452	Myalgia	ND	N
458	Joint pain	ND	N
459		ND	N
465		ND	N
466	Joint pain	ND	N
469			P
470			N

478		ND	N
487	Neurocognitive, Fatigue	ND	N
488	Neurocognitive, Neurological	ND	P
489	Joint pain, Fatigue	ND	P
490		ND	P
491		ND	N
492	Fatigue	ND	P
493	Neurocognitive	ND	N
495	Neurocognitive	ND	P
497		ND	P
498	Neurocognitive	ND	N
499		ND	N
500		ND	N
501	Joint pain	ND	P

1. Casjens SR, Fraser-Liggett CM, Mongodin EF, Qiu WG, Dunn JJ, Luft BJ, Schutzer SE: **Whole genome sequence of an unusual Borrelia burgdorferi sensu lato isolate.** *J Bacteriol* 2011, **193:**1489-1490.
2. Stanek G, Reiter M: **The expanding Lyme Borrelia complex--clinical significance of genomic species?** *Clin Microbiol Infect* 2011, **17:**487-493.
